# Compartmentalization of cerebrospinal fluid inflammation across the spectrum of untreated HIV-1 infection, central nervous system injury and viral suppression

**DOI:** 10.1371/journal.pone.0250987

**Published:** 2021-05-13

**Authors:** Magnus Gisslen, Sheila M. Keating, Serena Spudich, Victor Arechiga, Sophie Stephenson, Henrik Zetterberg, Clara Di Germanio, Kaj Blennow, Dietmar Fuchs, Lars Hagberg, Philip J. Norris, Julia Peterson, Barbara L. Shacklett, Constantin T. Yiannoutsos, Richard W. Price

**Affiliations:** 1 Department of Infectious Diseases, Institute of Biomedicine, Sahlgrenska Academy, University of Gothenburg, Gothenburg, Sweden; 2 Department of Infectious Diseases, Region Västra Götaland, Sahlgrenska University Hospital, Gothenburg, Sweden; 3 Vitalant Research Institute (formerly Blood Systems Research Institute), San Francisco, CA, United States of America; 4 Department of Neurology, Yale University School of Medicine, New Haven, CT, United States of America; 5 Department of Neurology, University of California San Francisco, San Francisco, CA, United States of America; 6 Department of Psychiatry and Neurochemistry, Institute of Neuroscience and Physiology, The Sahlgrenska Academy at the University of Gothenburg, Gothenburg, Sweden; 7 Clinical Neurochemistry Laboratory, Sahlgrenska University Hospital, Mölndal, Sweden; 8 Department of Neurodegenerative Disease, UCL Institute of Neurology, Queen Square, London, United Kingdom; 9 UK Dementia Research Institute at UCL, London, United Kingdom; 10 Institute of Biological Chemistry, Innsbruck Medical University, Innsbruck, Austria; 11 Department of Medical Microbiology and Immunology, University of California Davis, Davis CA, United States of America; 12 Department of Biostatistics, Indiana University R.M. Fairbanks School of Public Health, Indianapolis, IN, United States of America; University of Torino, ITALY

## Abstract

**Objective:**

To characterize the evolution of central nervous system (CNS) inflammation in HIV-1 infection applying a panel of cerebrospinal fluid (CSF) inflammatory biomarkers to grouped subjects representing a broad spectrum of systemic HIV-1 immune suppression, CNS injury and viral control.

**Methods:**

This is a cross-sectional analysis of archived CSF and blood samples, assessing concentrations of 10 functionally diverse soluble inflammatory biomarkers by immunoassays in 143 HIV-1-infected subjects divided into 8 groups: untreated primary HIV-1 infection (PHI); four untreated groups defined by their blood CD4+ T lymphocyte counts; untreated patients presenting with subacute HIV-associated dementia (HAD); antiretroviral-treated subjects with ≥1 years of plasma viral suppression; and untreated elite controllers. Twenty HIV-1-uninfected controls were included for comparison. Background biomarkers included blood CD4+ and CD8+ T lymphocytes, CSF and blood HIV-1 RNA, CSF white blood cell (WBC) count, CSF/blood albumin ratio, CSF neurofilament light chain (NfL), and CSF t-tau.

**Findings:**

HIV-1 infection was associated with a broad compartmentalized CSF inflammatory response that developed early in its course and changed with systemic disease progression, development of neurological injury, and viral suppression. CSF inflammation in untreated individuals without overt HAD exhibited at least two overall patterns of inflammation as blood CD4+ T lymphocytes decreased: one that peaked at 200–350 blood CD4+ T cells/μL and associated with lymphocytic CSF inflammation and HIV-1 RNA concentrations; and a second that steadily increased through the full range of CD4+ T cell decline and associated with macrophage responses and increasing CNS injury. Subacute HAD was distinguished by a third inflammatory profile with increased blood-brain barrier permeability and robust combined lymphocytic and macrophage CSF inflammation. Suppression of CSF and blood HIV-1 infections by antiretroviral treatment and elite viral control were associated with reduced CSF inflammation, though not fully to levels found in HIV-1 seronegative controls.

## Introduction

Infection of the central nervous system (CNS) is a nearly universal facet of untreated human immunodeficiency virus type 1 (HIV-1) infection and is accompanied by increased CNS immune activation and inflammation with frequent cerebrospinal fluid (CSF) lymphocytic pleocytosis and increased concentrations of a variety of inflammatory biomarkers [1–11]. Though long recognized, the CNS inflammatory state has not yet been systematically characterized in relation to the full evolving spectrum of systemic and CNS disease progression and treatment.

CNS HIV-1 infection develops very early in systemic infection [12–15] and continues throughout its untreated course, allowing detection of HIV-1 RNA in CSF of almost all untreated people living with HIV (PLWH) [6, 16–18], with the notable exception of elite viral controllers [19, 20]. During untreated chronic infection the concentration of CSF HIV-1 RNA is generally about one-tenth that of plasma, though with considerable individual variation and two general exceptions—during both primary infection and the late stage of systemic progression when blood CD4+ T cell counts have fallen below 50 cells/μL the ratio of CSF to plasma HIV-1 RNA is often wider, nearing a mean of 1:100 [6, 18, 21].

Systemic disease progression is also accompanied by evolution in the genetic and phenotypic characteristics of CNS infection with both shared and divergent HIV-1 populations when compared to those in plasma [22–25]. During early infection, CSF HIV-1 may be predominantly sustained in CD4+ T-lymphocytes (T-tropic) with CSF HIV-1 populations genetically similar to those circulating in blood [26], presumably as a result of CD4+ T-cell traffic that carries both infected and uninfected target CD4+ T cells into the CNS [26, 27]. Anatomically, this early infection may be located predominantly within the leptomeninges [28–30]. However, as systemic disease progresses, viral populations in the CNS may increasingly diverge from those of plasma, becoming *compartmentalized*, and also may more frequently favor replication in macrophages and related myeloid cells (M-tropic) [24, 31]. Finally, in the setting of advanced systemic infection, frank HIV-1 encephalitis may cause severe brain dysfunction and a syndrome that was initially termed subacute encephalitis [32] and AIDS dementia complex (ADC) [33, 34], and is now encompassed within the term HIV-associated dementia (HAD) [35]. Through these different stages of systemic and CNS disease progression and suppression, the character of CSF inflammation may also vary.

While primary systemic infection and the early phases of chronic CNS infection are most often asymptomatic, they may not always be neurologically innocent. Neurological dysfunction and evidence of CNS injury have been documented during the first year of infection [15], while milder or subclinical brain injury may develop in a more substantial number of PLWH over the course of chronic infection in the absence of frank HAD [36]. The latter may manifest as mild impairment on quantitative neuropsychological testing with little or only mild impact on daily living as codified in definitions of asymptomatic neurocognitive impairment (ANI) and minor neurocognitive disorder (MND) [35].

Measurement of CSF neurofilament light chain protein (NfL) provides a sensitive objective biomarker of ongoing neuronal injury, indicating *active* CNS disease, and complements quantitative neuropsychological test documentation of cognitive-motor impairment that may reflect the cumulative effects of both active and inactive (*legacy*) HIV-related and non-HIV-related CNS injury [37, 38]. CSF NfL elevations are particularly notable in patients presenting with subacute HAD [39, 40], but are also found in some with milder or subclinical injury that increases in frequency as systemic disease advances, particularly when blood CD4+ T-cells fall below 200 cells per μL [41].

As with its systemic counterpart, combination ART has a major impact on CNS HIV-1 infection, so that individuals with systemic viral suppression with reduction of plasma HIV-1 RNA levels to below standard clinical detection limits usually also exhibit effective CSF HIV-1 RNA suppression [42–45]. Indeed, even when assessed by very sensitive assays, HIV-1 RNA detection is characteristically less frequent and measured at lower levels in CSF than plasma [20, 45, 46]. However, there are also notable exceptions to CNS virological efficacy: *CSF HIV-1 escape* in which CSF HIV-1 RNA levels exceed those of plasma, either with clinical impact (*neurosymptomatic CSF escape*) [47, 48] or not (*asymptomatic CSF escape*) [49], or in the presence of another CNS infection (*secondary CSF escape*) [50]. While these escape settings were not included in this study, their inflammatory profiles warrant further study [51].

Like systemic HIV-1 infection [52, 53], CNS infection is accompanied by local inflammation that is reduced in the face of effective ART [6]. For example, levels of CSF neopterin, an extensively studied inflammatory biomarker in HIV infection [8], fall very rapidly after ART initiation [54], and CSF pleocytosis characteristically also resolves, though generally more slowly [6]. However, CNS inflammation does not always fully ‘normalize’ with suppressive treatment, and CSF neopterin and T-cell activation may persist at levels above those of HIV-uninfected controls despite ‘undetectable’ CSF HIV-1 RNA [55, 56]. Even very low levels of CSF HIV-1 RNA may associate with higher levels of local inflammation than more complete CSF viral suppression [46, 57].

While CNS immune and inflammatory reactions may participate in local viral control, they also may contribute to brain injury [58, 59]. In the absence of direct HIV-1 infection of neurons, immune and inflammatory reactions are speculated to be a key source of signals and toxins responsible for neuronal dysfunction and death [60]. Thus, in the broad sense, neuronal injury and neurological dysfunction in HIV-1 infection likely have important immunopathological components in which the integrity of uninfected neurons is altered by events in neighboring cells, particularly macrophages and likely astrocytes, though the exact contributions of individual viral-coded and inflammatory molecules to *in vivo* injury remain largely inferential [9, 60–62]. Further, it is possible that ‘residual’ inflammation detected in treated individuals contributes to ongoing (albeit subclinical) brain injury and neuropsychological test performance impairment despite viral suppression [63]. While the factors driving this continuous inflammation are not firmly established, a persistent CNS HIV-1 reservoir may be an important factor [64–67].

While a number of the components of local CNS inflammation have been documented in different cohorts or case series, the overall character of evolving CNS inflammation has not yet been clearly delineated in relation to the stages of systemic infection, CNS injury and antiretroviral treatment (ART) [8, 21, 41, 54, 57].

To begin to address this deficiency, we undertook this cross-sectional exploration of a set of subject groups encompassing the main stages of HIV-1 systemic infection and CNS injury using a panel of 10 functionally diverse, soluble inflammatory biomarkers measured in CSF and blood with major attention to the former. With these measurements we examined questions related to the compartmentalization of CSF inflammation and the evolving course of inflammation from early (primary) infection, through declining CD4+ T-lymphocyte counts and the development of HIV-associated dementia (HAD), and after treatment or during endogenous viral suppression.

## Materials and methods

### Study design

This cross-sectional study was an extension of an earlier published study using a set of archived CSF and matching blood samples derived from two cohorts: one in San Francisco, California, USA and the other in Gothenburg, Sweden [41]. The samples and related background data were all obtained between 1994 and 2010 within the context of research protocols approved by the institutional review boards of the two study sites (UCSF IRB Protocol 10–0727 and Gothenburg Ethical Commmitttee DNr 0588–01); written informed consent was obtained from all subjects. CSF and matching blood samples, along with data related to background HIV-1 and neurological status, were included from 8 predefined HIV-infected subject groups based on time from initial infection, T-cell stratum, neurological presentation, and viral suppression as previously described [41].

### CSF and blood sampling

CSF was obtained according to standard protocols as previously described [6, 39, 68, 69]. Subjects also underwent phlebotomy for concurrent blood sampling, along with general medical and neurological assessments at the study visit as previously described [70]. CSF was placed immediately on wet ice and subsequently subjected to low-speed centrifugation to remove cells, aliquoted and stored within 2 hours of collection at ≤-70°C until the time of HIV-1 RNA and biomarker assays. Blood was collected either in EDTA or as serum, aliquoted and stored in parallel with CSF for later batch assays.

### Clinical evaluations

All subjects underwent routine clinical bedside screening for symptoms or signs of CNS opportunistic infections or other conditions that might impact CSF biomarker concentrations; individuals with CNS opportunistic infections or other conditions confounding these analyses were omitted. Most of these subjects were studied before publication of the more formal Frascati criteria [35] and were diagnosed with ADC stages 2–4 [71] and met American Academy of Neurology criteria in place at the time [72]; retrospectively, they also met the functional criteria for the Frascati diagnosis of HAD without the requisite extensive formal neuropsychological assessment, so this more contemporary term was used to encompass this group. The designation as ADC/HAD was based on clinicians’ assessment at the time of diagnostic presentation, characteristically after subacute onset and progression of cognitive and motor symptoms and signs. The HIV-uninfected controls were volunteer subjects evaluated in San Francisco and drawn from the same overall population at San Francisco General Hospital as the PLWH cared for in this center as previously described [41]. Subjects in San Francisco also underwent brief quantitative neurological performance testing to derive an aggregate normalized Z score on four tests (QNPZ-4 averaging the normalized scores on grooved pegboard, digit symbol, finger tapping, and timed gait tests) [15, 41, 73].

### Background laboratory methods

HIV-1 RNA levels were measured in cell-free CSF and plasma at each site using the ultrasensitive Amplicor HIV Monitor assay (versions 1.0 and 1.5; Roche Molecular Diagnostic Systems, Branchburg, NJ), Cobas TaqMan RealTime HIV-1 (version 1 or 2; Hoffmann-La Roche, Basel, Switzerland) or the Abbott RealTime HIV-1 assay (Abbot Laboratories, Abbot Park, IL, USA). All recorded viral loads that were below 40 copies per mL were standardized to an assigned ‘floor’ value of 39 copies per mL for descriptive purposes. Each study visit included assessments by local clinical laboratories using routine methods to measure CSF white blood cell (WBC) count, CSF and blood albumin in order to assess blood-brain barrier integrity, and blood CD4+ and CD8+ T lymphocyte counts by flow cytometry. CSF neurofilament light chain protein (NfL) concentration was measured using a sensitive sandwich method (NF-light^®^ ELISA kit, UmanDiagnostics AB, Umeå, Sweden) and analyzed in a single run in the Laboratory of Neurochemistry at the University of Gothenburg by board-certified laboratory technicians blind to clinical data using a single batch of reagents for each assay; intra-assay coefficients of variation were below 10% for all analyses. To compare NfL values across all groups, we calculated age-adjusted NfL values for 42 years of age as outlined by Yilmaz and colleagues, and compared to the 42 year-old upper limit of normal of 757.8 ng/L [38]. The CSF NfL results have been previously reported [41]. CSF t-tau levels, also previously reported, were measured by published methods [41].

The background demographic and salient HIV-related laboratory data of the study subject groups are summarized in **Table 1**. These have been described in an earlier study of this sample reporting analysis of neural biomarkers [41]. The number of subjects (N) initially targeted for each group was 20, but exceptions included PHI (N = 24), treated-suppressed (N = 19), HAD (N = 12), and elites (N = 8), with the latter two group deficiencies determined by limited availability. The median nadir blood CD4+ T-cell count of the treatment-suppressed group was 60 (IQR 12–220) cells/μL, while for other groups the nadir CD4+ counts were equal to or near the documented visit values.

**Table 1:** Background characteristics of the 9 subject groups.

	Units	HIV negative	PHI	NA, CD4 >350	NA, CD4 200–349	NA, CD4 50–199	NA, CD4 <50	HAD	RX suppressed	Elites
**VARIABLE**						N				
***Subjects***	Total per group	20	24	20	20	20	20	12	19	8
***Site***	Ratio SF:GOT	20:0	19:5	12:8	12:8	12:8	11:9	6:6	11:8	8:0
***Gender***	Ratio M:F	20:0	24:0	16:4	15:5	17:3	18:2	12:0	16:3	5:3
		median (25–75%)								
***Age (years)***	years	36 (29–48)	34 (28–43)	42 (36–44)	45 (35–53)	43 (38–54)	42 (38–49)	42 (38–47)	43 (38–49)	49 (42–55)
***Time since HIV diagnosis***	"		0.14 (0.10–0.19)	2 (0.7–12)	4 (0.7–14)	1 (0.7–12)	1 (0.1–13)	2 (4–8)	2 (0.0–8)	19 (14–23)
***Background Biomarkers***										
***Visit CD4***	cells/μL	762 (688–988)	559 (401–749)	491 (409–572)	240 (222–306)	122 (96–145)	20 (8–39)	55 (35–160)	548 (283–770)	869 (721–1070)
***Visit CD8***	"	534 (351–683)	1200 (708–1623)	909 (694–1142)	827 (694–1086)	760 (497–930)	495 (285–684)	650 (307–976)	757 (620–1094)	702 (401–828)
***Nadir CD4***	"			320 (235–370)	220 (60–285)	110 (70–177)	15 (4–26)	20 (14–105)	60 (12–220)	
***CSF WBC***	"	1 (1–3)	6 (3–13)	7 (3–9)	6 (3–14)	4 (1–9)	0 (0–1)	6 (1–18)	1 (0–2)	2 (2–2.8)
***HIV plasma RNA***	log_10_ copies/mL		4.6 (4.2–5.5)	4.1 (3.4–4.6)	4.8 (4.2–5.3)	4.8 (4.5–5.1)	5.4 (4.5–5.9)	5.3 (4.8–5.7)	1.6 (1.6–1.6)*	1.6 (1.6–1.6)*
***HIV CSF RNA***	"		2.7 (1.8–3.4)	3.2 (2.5–3.6)	4.1 (3.4–4.6)	3.8 (3.5–4.4)	3.1 (2.3–3.8)	3.6 (2.7-.0)	1.6 (1.6–1.6)*	1.6 (1.6–1.6)*
***Plasma*:*CSF difference***	"		2.1 (1.7–2.3)	1.0 (0.1–1.6)	0.6 (0.4–1.4)	1.0 (0.6–1.5)	2.1 (1.6–3.0)	0.8 (0.4–2.1)	0 (0–0)	0 (0–0)
***CSF*: *blood Albumin***	ratio	4.3 (3.4–6.2)	5.4 (4.5–7.4)	5.3 (3.9–6.4)	5.1 (4.1–7.4)	5.4 (3.6–7.5)	4.4 (3.7–5.4)	9.8 (5.8–12.8)	5.1 (4.5–6.4)	4.4 (3.9–6.0)
***Neural Injury Biomarkers***										
***CSF NFL***	ng/L	364 (276–479)	509 (404–975)	487 (379–584)	567 (430–685)	611 (498–1243)	1861 (715–4600)	14915 (2576–39738)	620 (430–710)	574 (534–751)
***CSF t-tau***	"	201 (147–238)	215 (172–294)	203 (150–273)	190 (127–280)	233 (138–308)	250 (157–368)	603 (323–1275)	187 (130–240)	195 (172–294)
***QNPZ-4***	Z score	-0.10 (-0.55–0.16)	-0.16 (-0.74–0.04)	-0.07 (-0.63–0.40)	-0.16 (-0.67–0.59)	-0.45 (-1.80–0.11)	-1.42 (-12.58- -0.10)	-2.42 (-5.22- —1.32)	0.78 (0.04–1.11)	-1.08 (-1.52- -0.28)

*These log_10_ values are equivalent to the assigned ’floor’ value of 39 copies/Ml for all values below 40 copies/mL.

### Inflammatory biomarker measurements

We included immunoassays of 10 CSF and blood soluble inflammatory biomarkers selected on the basis of previous studies or theoretical interest as representing different relevant components of the CNS inflammatory response [10, 74–80]. CSF and blood concentrations of TNFα, MMP9, CXCL10 (IP-10), sCD14, sCD163, sVCAM-1, CCL2 (MCP-1), IL6, and TIMP1 used multiplex bead array or ELISA methods at the Blood Systems Research Institute (now Vitalant), San Francisco (see biomarker Glossary below). Five different assays were used including Milliplex high sensitivity cytokine multiplex (TNFα and IL6); Millipore, Billerica MA), Milliplex standard sensitivity cytokine multiplex (IP-10 and MCP-1), Milliplex cardiovascular disease panel 1 (MMP9 andsVCAM1), Milliplex TIMP Panel 2 (TIMP1), sCD163 and sCD14 ELISA (R&D Systems, Minneapolis MN). Results were acquired by Luminex 200, analyzed by Bioplex 6.1 and compiled using Bioplex Datapro (Bio-Rad, Hercules CA). Blood measurements were all performed using plasma except sCD163 which used serum. CSF and blood neopterin concentrations were measured by a commercially available enzyme-linked immunosorbent assay (Neopterin RIA and EIA, BRAHMS, Hennigsdorf, Germany) in Innsbruck, Austria, also using a single reagent batch; these results have also been previously reported [41] but are included in this report for comparison with the other nine inflammatory biomarkers. This study was structured as an extension of our earlier one [41] using the same overall sample set. However, because some of the samples for CSF or blood assays had been exhausted, the number assessed for the inflammatory biomarkers in some cases was reduced; likewise, some of the background biomarkers were not available for all subjects. Because we did not modify the overall sample selection, the total numbers are listed in **Table 1**, and where sample numbers were reduced for from these numbers for particular assessments, the final number assayed are indicated in the main Figure legend.

### Statistics and analysis plan

Biomarker associations were analyzed across the entire sample set using Spearman’s rank correlation, graphically presented as a heat map. Comparison of biomarker concentrations between subject groups to address selected *a priori* questions used non-parametric methods, including Mann-Whitney to compare two groups and Kruskal-Wallis with Dunn’s *post hoc* test to compare three or more groups. For some of these, the results of comparisons are listed in the text or augmented by graphical isolation of these group comparisons. These statistics and graphs were performed with GraphPad Prism (versions 8, up to version 8.4.3, GraphPad Software, San Diego, USA). Linear and quadratic orthogonal (“appropriate”) contrasts across the four NA groups explored trends across using ANOVA were performed using SAS version 9.4 (SAS Institute, Cary, NC).

## Results and discussion

In this combined section of Results and Discussion, we will first outline the background characteristics of the cross-sectional sample, follow with a description of the overall results of biomarker measurements and analysis of CSF biomarker associations, and finally explore comparisons of selected subject group differences and their implications for understanding the evolution of CNS inflammation and its response to, and potential role in, CNS HIV-1 infection and CNS injury over the course of disease. Our main emphasis will be on the CSF inflammatory biomarker findings, while their blood counterparts will be considered mainly among the features of the background context to CNS inflammation.

### Overall results: CSF (and blood) inflammatory biomarkers across the cohort

**Fig 1:**
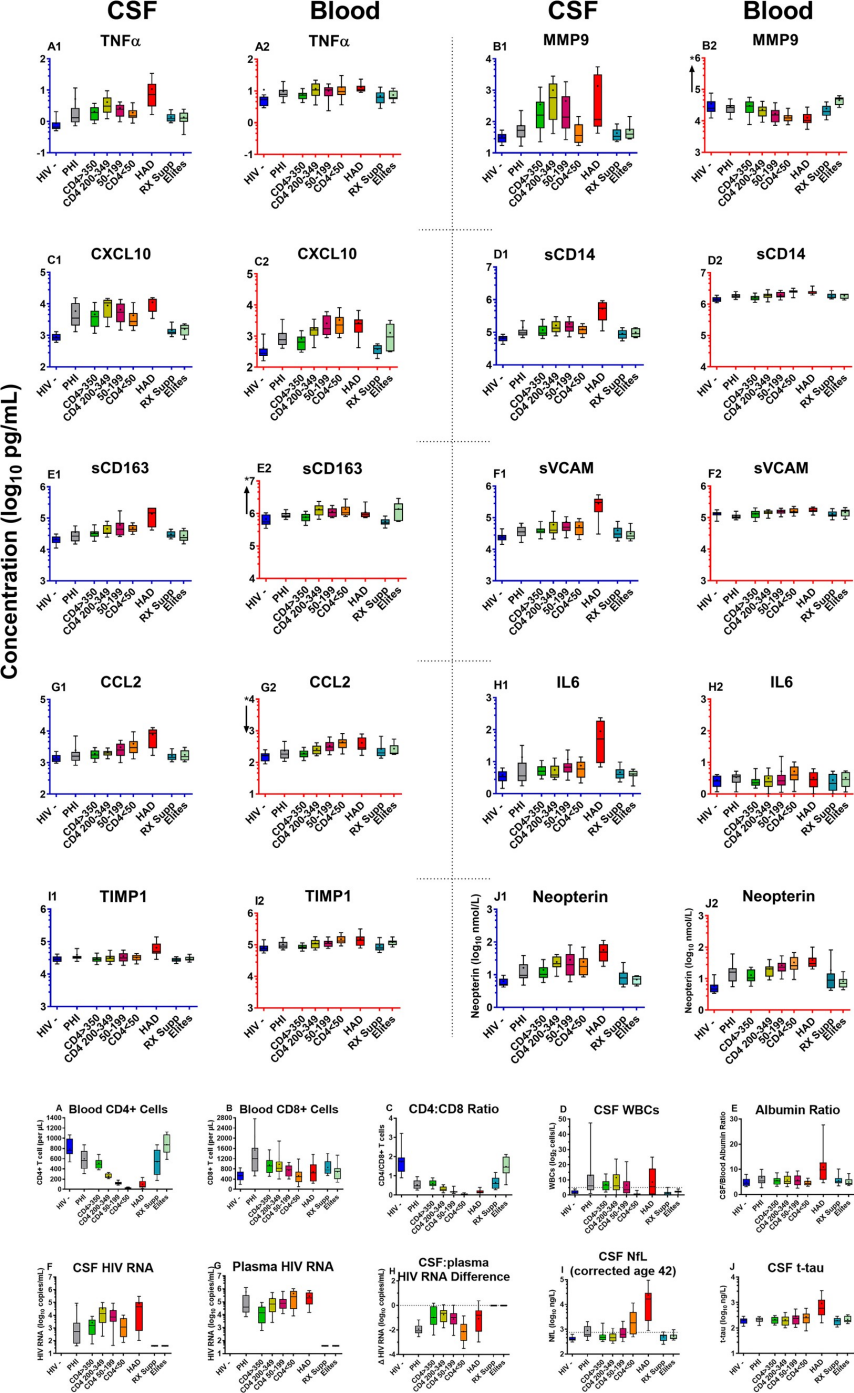
CSF and blood inflammatory biomarkers across the 9 subject groups. ***a*. *CSF and blood inflammatory biomarkers***. The panels plot CSF and blood biomarker results in adjacent pairs (e.g., A1 and A2 showing TNFα concentrations in CSF and plasma). For each pair, the CSF concentrations are shown to the left with blue axes and the blood concentrations to their right with red axes. Each biomarker is presented as log_10_ concentrations over a 3 log_10_ range, with the absolute ranges depending on the concentrations in the assessed fluid. Where the plotted ranges differed between CSF and blood for a given biomarker, an “*” and arrow near the blood biomarker’s Y axis shows the shift in axis up or down. Boxes show median and interquartile range with “+” denoting means; whiskers designate 10 and 90^th^ percentiles. Results are discussed in the text. Inflammatory biomarker concentrations in this and subsequent figures are in log_10_ pg/mL except neopterin in log_10_ nmol/L. The total number of subjects in each group are listed in **Table 1** but the number assayed for CSF and blood inflammatory markers were not fully concordant because of sample exhaustion in some cases. The assayed group numbers for CSF and for blood (in parentheses) analyzed for inflammatory markers were: HIV- 20 (20); PHI 24 (24); CD4 >350 20 (20), CD4 200–349 20 (20), CD4 50–199 20 (20); CD4 <50 19 (20); HAD N = 10 (12); Rx Suppressed 19 (18); Elite controllers 8 (7). ***b*. *Background biomarkers***. To facilitate visual comparison with the soluble biomarkers assessed for this study, these 10 graphs present the salient background findings in the same color-coded format. These data were previously published for this cohort [41]. The actual assayed group numbers are the same as the total group numbers in **Table 1** with a few exceptions (actual numbers in parentheses): blood CD4+ T cells for HAD (11); blood CD8+ cells and CD$/CD8 ratio for PHI (23) and HAD (11); Albumin ratio for HAD (11); CSF tau for Rx Supp (18).

As shown in **Fig 1A** depicting the concentrations of each of the 10 biomarkers in CSF and plasma across the 9 subject groups, HIV-1 infection induced an enhanced inflammatory state in CSF (and blood) across its full spectrum. The complete data set for the study results in **S1 Table** (deposited in Dryad: https://doi.org/10.7272/Q61R6NRZ).

Among the major features of the broad and robust CSF inflammatory response were: ***1***. CSF biomarker concentrations varied in relation to their blood counterparts in absolute terms at ‘baseline’ (HIV negative) and also in relative terms over the spectrum of HIV-1 disease; ***2***. the CSF inflammatory biomarkers exhibited higher concentrations in the untreated HIV-1-infected groups than in the uninfected controls in CSF (and in blood with the exception of MMP9), though the patterns of change across the groups differed among the biomarkers; ***3***. high CSF but not blood levels of all the markers were found in the HAD group; and ***4***. viral suppression by ART or by elite control reduced the CSF biomarker elevations, though not always to levels found in the HIV-negative controls. ***5***. Similarities in the patterns of some of the CSF biomarker changes allowed them to be grouped into hypothetical *pathogenetic vectors* representing more general inflammatory processes during evolution of untreated infection.

In order to dissect the elements of the CSF inflammatory features of HIV-1 infection, we will first examine the broad correlations among the 10 biomarkers across the entire sample. We follow this with more focused attention on selected subject groups and group comparisons to understand inflammatory transitions during disease progression and their associations with the characteristics of CNS infection and neurological injury.

### Correlations among the CSF and blood inflammatory biomarkers and the background biomarkers across the entire sample

**Fig 2:**
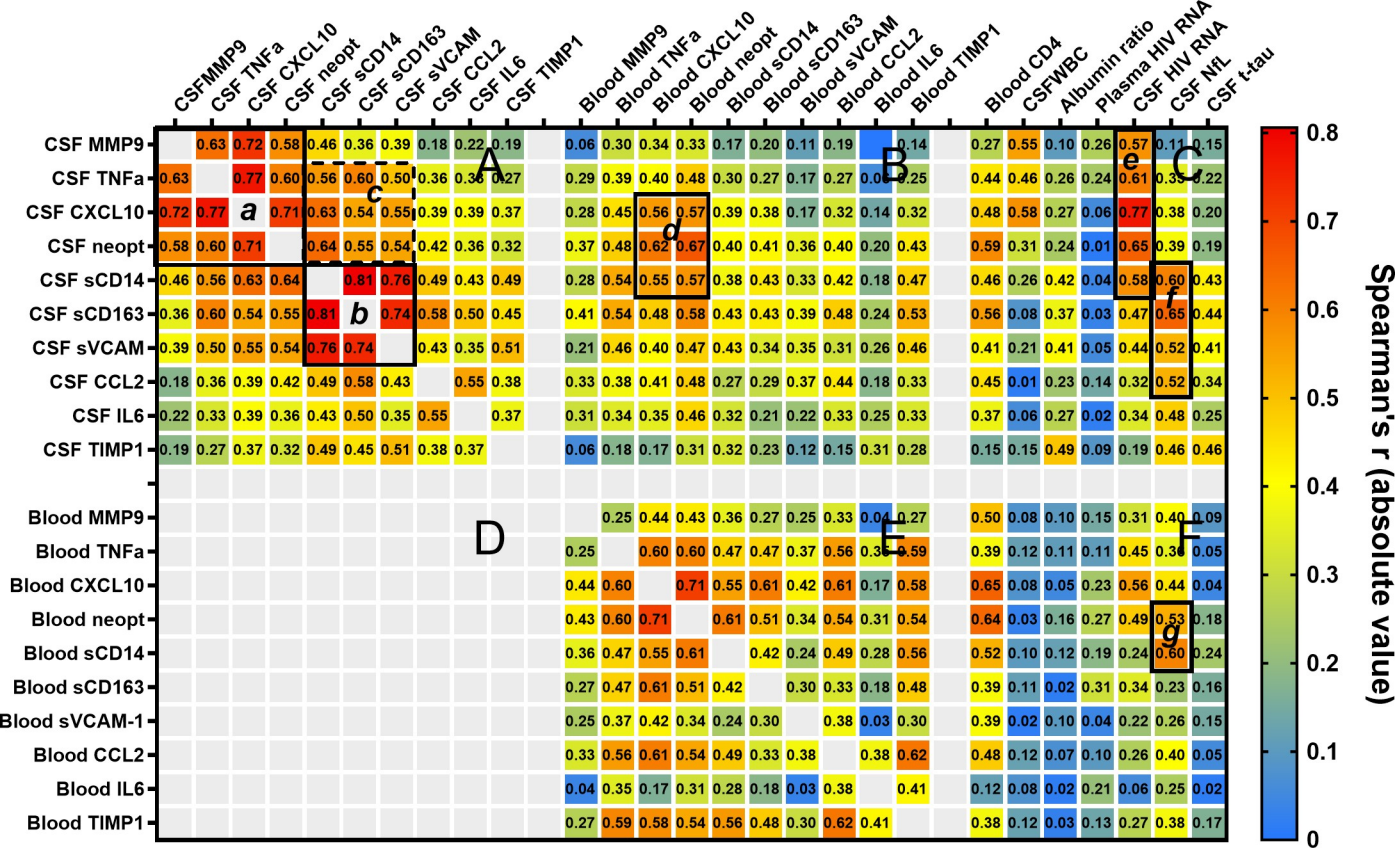
Correlations among the CSF and blood biomarkers and with selected background biomarkers. This blue-yellow-red heat map diagrams the Spearman’s correlation coefficient ***r*** across the entire data set expressed as absolute values and provides an overview of variable correlations. The absolute values are used to simplify comparison of positive and negative correlations, the latter involving blood CD4+ T cell counts and blood MMP9 concentrations which generally varied inversely with the other markers. The color scale provides a visual index while the *r* values are provided in each cell, mapping the relationships among the CSF and blood biomarker concentration across the entire sample. The large panels plot the Spearman’s r: among the CSF (**A**) and blood (**E**) inflammatory biomarkers; between the CSF and blood inflammatory biomarkers (**B);** and between the CSF and blood inflammatory biomarkers and seven salient background biomarkers (**C** and **F**). Panel **D** is blank for visual simplicity since it would show the same matrix as **B**, flipped along a diagonal axis. The smaller outlined boxes (**a-g**) highlight some of the interesting biomarker relationships of interest as discussed in the text.

To explore the relationships among the 10 CSF inflammatory biomarkers and their associations with both these same biomarkers in blood and the salient background biomarkers, we examined biomarker inter-correlations across the entire sample irrespective of group designation. The inclusion of the broad range of subject groups in this study provided a wide sampling of the HIV-1 systemic and CNS disease spectrum for this overall analysis. The heat map in **Fig 2** presents the Spearman’s correlation coefficients and a visual overview of the biomarker associations.

In general, the inflammatory biomarker concentrations were extensively inter-correlated, consistent with the broad CNS and systemic immune activation and inflammation associated with HIV-1 infection. Notably, some of the biomarkers aggregated into discernible group patterns. For example, CSF MMP9, TNFα, CXCL10 and neopterin exhibited high levels of inter-correlations, with CXCL10 showing the highest correlations with the other three of these markers (highlighted by the **Box *a*** drawn in the upper left corner of **Panel A** of **Fig 2**), suggesting relationships in their biological activity across HIV-1 progression. Similarly, CSF sCD14, sCD163 and sVCAM1 exhibited very high inter-correlations (highlighted by the drawn **Box *b*** in the middle of **Panel A** diagonally below the first box); this indicates a second group of biomarkers with related responses across the full spectrum of disease. Moreover, these two drawn boxes of correlating biomarkers exhibited additional overlapping correlations, though excluding MMP9 (outlined by the third **Box *c*** to the right of ***a*** and above ***b***). These correlations highlight associations or interactions among 7 of the CSF biomarkers that form two overlapping clusters.

These biomarker groups are of additional interest because of their further correlations with some of the background biomarkers. Notably, the CSF HIV-1 RNA concentrations correlated highly with the CSF biomarkers highlighted in **Box *a***—most intensely with CSF CXCL10—and with sCD14 from **Box *b*** designated by the outlined partial **Column *e*** within **Panel C**. CSF NfL correlated most strongly with those of **Box *b***, particularly with CSF sCD163, but also with sCD14, sVCAM-1 and CCL2 as outlined by the partial **Column *f***—and much more weakly with those of the ***a*** group. There was thus a dissociation of the profiles of inflammation associated with CSF HIV-1 infection, on the one hand, and axonal injury, on the other, with sCD14 shared to some extent. The CSF WBC count correlated most clearly with CSF CXCL10 and MMP9. CSF TIMP1 showed the highest correlation with the CSF/blood albumin ratio, in keeping with previous observations of this marker and blood-brain barrier dysfunction [81, 82]. CSF t-tau, which is a less sensitive marker of neuronal injury in HIV-1 infection than CSF NfL, generally associated with the same CSF inflammatory biomarkers as NfL, though to a lower degree. It also included correlation with CSF TIMP1, presumably related to the damaged blood-brain barrier or other cellular changes present in the HAD group.

Individual blood biomarker concentrations generally showed only relatively weak correlations with their CSF counterparts, with the notable exceptions of CXCL10 and neopterin (**Fig 2 Panel B Box *d***). Blood concentrations of both these biomarkers correlated relatively strongly with their CSF counterparts and almost as strongly with CSF sCD14 and sCD163 suggesting that their blood concentrations—and the systemic inflammatory state that they indicate—were associated with CNS inflammation. These two blood markers correlated strongly with CD4 count, plasma HIV-1 RNA and somewhat less strongly to CSF RNA (**Fig 2 Panel F**). Additionally, among the blood markers, sCD14 and neopterin showed strong correlations with CSF NfL (**Panel F, Column *g***), at a level similar to that of the CSF markers correlating with injury, suggesting a systemic inflammatory profile predisposing to CNS injury. While these CSF and blood inflammatory patterns do not establish cause or effect, they suggest pathobiological links between components of CNS and systemic inflammation with axonal injury.

### Stages of infection: Subject group comparisons

For a more focused view of the changing CSF inflammatory characteristics accompanying systemic disease progression, development of neuronal injury and viral suppression, we compared the concentrations of the CSF biomarkers among selected subject groups. These isolated group comparisons were selected *a priori* to address individual questions.

#### HIV seronegative controls: Relation of CSF to blood inflammatory biomarker concentrations

Before comparing the changes in inflammatory biomarkers during infection, it is pertinent to examine CSF concentrations in the HIV-uninfected controls which varied considerably among the 10 biomarkers both in absolute concentrations and in relation to their blood counterparts. Comparison of mean CSF and blood inflammatory biomarker concentrations are shown in **Table 2**. These ratios of CSF-to-blood concentrations varied from nearly 1:1,000 (MMP9) to a nine-fold higher CSF than blood concentration (CCL2). Seven of these concentration differences were highly significant, the exceptions being TNFα, with high variability in the sample, and both neopterin and IL-6 with CSF concentrations that were close to those in blood.

**Table 2:** Comparison of CSF and blood concentrations of the inflammatory biomarkers in HIV-uninfected group. The ratios between CSF and blood concentrations of the 10 markers are listed in ascending order.

BIOMARKER	CSF		Blood		CSF:Blood ratio		CSF-blood difference
(in order of CSF:blood ratio)	Mean Value	Log_10_	Mean Value	Log_10_	Arithmatic ratio	Log_10_ difference	P value*
***MMP9***	33.95	1.531	35022	4.544	0.0010	-3.014	**<0.000001**
***sCD163***	21332	4.329	681256	5.833	0.0313	-1.504	**<0.000001**
***sCD14***	65864	4.819	1429301	6.155	0.0461	-1.336	**<0.000001**
***TNF*α**	0.8694	-0.061	10.93	1.039	0.0795	-1.099	0.091
***sVCAM***	25762	4.411	132556	5.122	0.1943	-0.711	**<0.000001**
***TIMP-1***	29329	4.467	83533	4.922	0.3511	-0.455	**<0.000001**
***Neopterin***	6.338	0.802	5.789	0.763	1.0948	0.039	0.621
***IL-6***	3.811	0.581	2.752	0.440	1.3848	0.141	0.067
***CXCL10***	898.6	2.954	419.1	2.622	2.1441	0.331	**0.000168**
***CCL2***	1466	3.166	163.9	2.215	8.9445	0.952	**<0.000001**

*P value from individual T tests comparing CSF and blood concentrations in HIV- group.

This analysis indicates that even in the absence of infection the CSF biomarkers are compartmentalized with a range of concentration relationships to blood. Thus, even in the absence of HIV-1 infection each of these inflammatory factors is ‘regulated’ in an individual manner, presumably by different levels of local production but perhaps also by variable kinetics of exchange between blood and CSF and cell uptake. They indicate that the ‘physiological’ role of these and other inflammatory molecules in the ‘baseline’ state is a worthy subject of further study [83, 84].

#### Primary infection: Onset of CNS inflammation and transition to chronic infection

Like CNS infection, CSF inflammation is clearly established during the first year of infection [13, 15, 85]. In this study we included 25 individuals with primary HIV infection (PHI), studied during the first year of infection. This PHI subjects were drawn from a larger group that we have described in more detail previously [13]. Based on laboratory biomarkers, clinical histories (particularly time of acute retroviral syndrome) and serologies at and before diagnosis, the median time from HIV-1 exposure in this group was 51 days (IQR 45–69; range 32–133 days, with the high value was a distinct outlier). Thus, these subjects were early in infection but not in the ‘hyperacute’ or ‘acute’ phases segregated by Fiebig stages 1-IV. They were also clearly pathobiologically different from those with chronic infection and higher CD4 counts This was perhaps most notably in their relatively higher blood and lower CSF HIV-1 RNA levels with resultant high plasma to CSF HIV-1 RNA ratios (**Fig 1B**). In an earlier study of a larger sample with PHI, we found that the albumin quotient was elevated in about 20% of subjects [86]; in the current study this proportion was lower, but still showed a small mean elevation above the HIV negative group, along with a similar small elevation in mean NfL.

**Fig 3:**
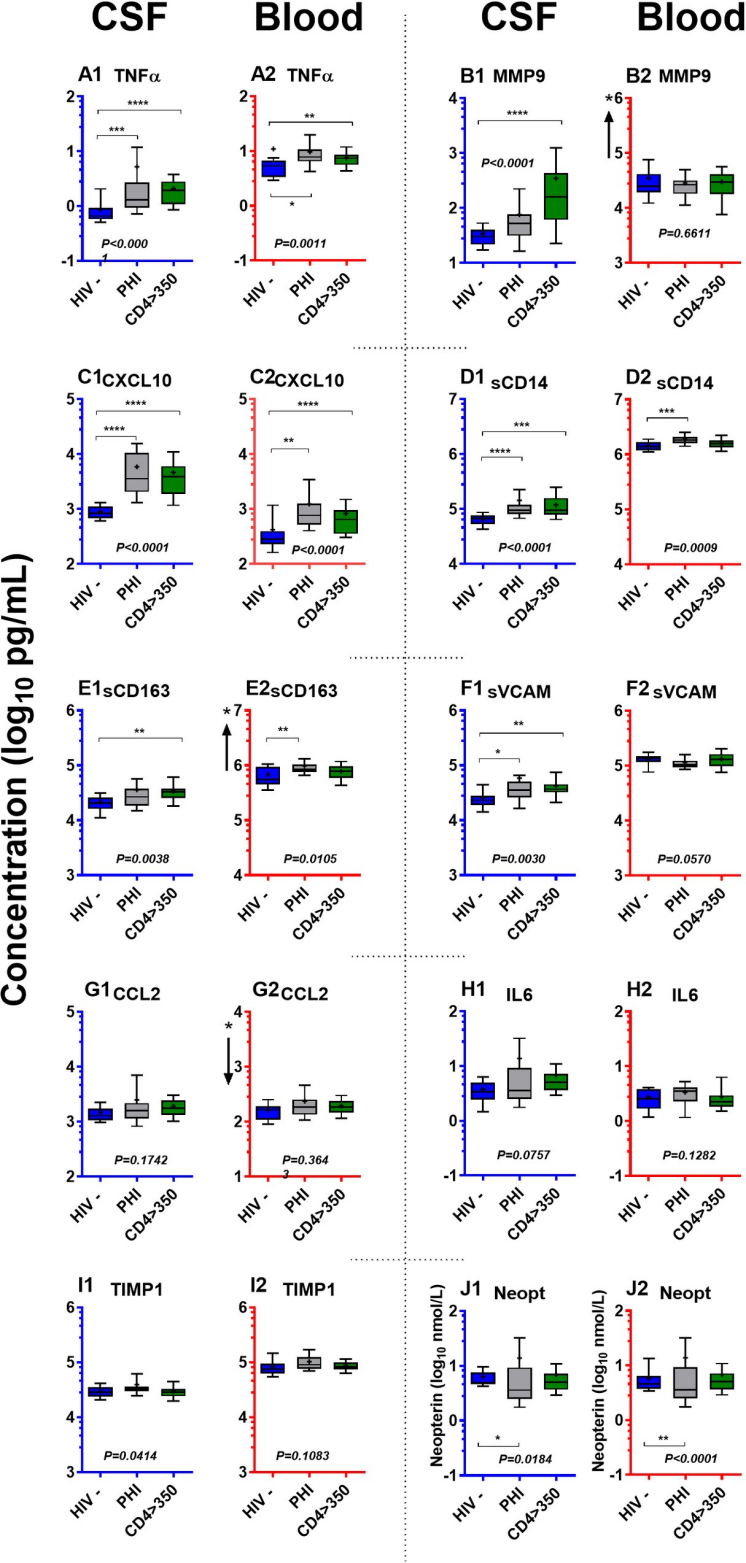
CSF and blood inflammatory biomarkers in PHI compared to HIV-uninfected and early chronic infection. These graphs use the same format, axes and unit scales as **Fig 1A**. but isolate three subject groups: HIV negatives, PHI and the NA group with >350 CD4+ blood T cells in order to examine the ‘transition’ during PHI from seronegative to early chronic infection. P values in italics give the overall Kruskal-Wallis ANOVA significance, while the horizontal bars show P values of individual *post hoc* testing using Dunn’s multiple comparison test for the three-group comparison: * P <0.05; ** P <0.01; *** P <0.001; and **** P <0.0001. This analysis is also outlined in **Table 3**. Since this figure is extracted from **Fig 1A**, the subject numbers are listed in the legend of that figure.

In order to examine the CSF (and blood) inflammatory reactions during this first year in more detail, including how they compared to uninfected individuals, on the one hand, and the early phase of chronic infection, on the other, we isolated comparisons of the biomarkers results in three subject groups: 1. uninfected HIV negative controls, 2. PHI and 3. HIV-1-infected NA group with highest CD4+ T-cell counts, >350 cells/μL, applying the Kruskal-Wallis test and Dunn’s multiple comparison test as shown in **Fig 3**.

The concentrations of all the inflammatory biomarkers were higher in CSF (and blood) during PHI than in the HIV-negative controls. These increases varied from 33% (TIMP-1) to 500% (TNFα and MMP9) of the HIV-negative level (**Table 3**). The median change was greater than a twofold increase, with the change reaching significance in this analysis for 4 of the 10 biomarkers: TNFα, CXCL10, sCD14 and neopterin (all with P <0.001) with a less notable significance for sVCAM (P <0.05).

**Table 3:** CSF biomarkers in early infection: comparisons of HIV negative, PHI and chronic infection with higher CD4 counts.

BIOMARKER	HIV negative CSF		PHI CSF		CD4 >350		PHI:HIV- Ratio			PHI:HIV- difference	>350:PHI Ratio			>350:PHI difference
(in order of CSF:blood ratio)	Mean Value	Log_10_	Mean Value	Log_10_	Mean Value	Log_10_	Arithmatic ratio	Percent change	Log_10_ difference	P value*	Arithmatic ratio	Percent change	Log_10_ difference	P value*
***TNF*α**	0.8694	-0.061	5.214	0.717	2.085	0.319	5.997	499.7%	0.778	**<0.001**	0.400	-60.0%	2.283	NS
***MMP9***	33.95	1.531	76.16	1.882	347.3	2.541	2.243	124.3%	0.351	NS	4.560	356.0%	1.118	NS
***CXCL10***	898.6	2.954	5860	3.768	4630	3.666	6.521	552.1%	0.814	**<0.0001**	0.790	-21.0%	-0.768	NS
***sCD14***	65864	4.819	143033	5.155	33927	4.531	2.172	117.2%	0.337	**<0.0001**	0.237	-76.3%	-2.155	NS
***sCD163***	21332	4.329	35855	4.555	33927	4.531	1.681	68.1%	0.226	NS	0.946	-5.4%	-1.555	NS
***sVCAM***	25762	4.411	58642	4.768	42607	4.629	2.276	127.6%	0.357	**<0.05**	0.727	-27.3%	-1.768	NS
***CCL2***	1466	3.166	2478	3.394	1913	3.282	1.690	69.0%	0.228	NS	0.772	-22.8%	-0.394	NS
***IL-6***	3.811	0.581	13.81	1.140	6.898	0.839	3.624	262.4%	0.559	NS	0.499	-50.1%	1.860	NS
***TIMP-1***	29329	4.467	39200	4.593	39200	4.593	1.337	33.7%	0.126	NS	1.000	0.0%	-1.593	NS
***Neopterin***	6.338	0.802	16.25	1.211	14.03	1.147	2.564	156.4%	0.409	**<0.05**	0.863	-13.7%	1.789	NS

*P values from Kruskal-Wallis test across three groups (HIV negative, PHI, and CD4 >350) and Dunn’s multiple comparison test.

These differences indicate that CNS inflammation develops within the first year of HIV-1 infection with changes in four CSF biomarkers showing the most substantial changes. Moreover, comparison of the PHI CSF biomarker concentrations to those in the >350 CD4 group with chronic infection show that most (8 of the 10) exhibit lower mean levels in the CD4 >350 group, emphasizing that the first year of infection exhibited a particularly enhanced inflammatory state that subsided somewhat after transition to chronic infection. Notably, this robust CSF inflammation during the first year of infection involved a group of biomarkers that also exhibited intercorrelations over the entire samples as discussed earlier and outlined in **Fig 2**.

#### Evolving CSF inflammation during CD4 decline in the NA subjects in the absence of overt HAD

**Fig 4:**
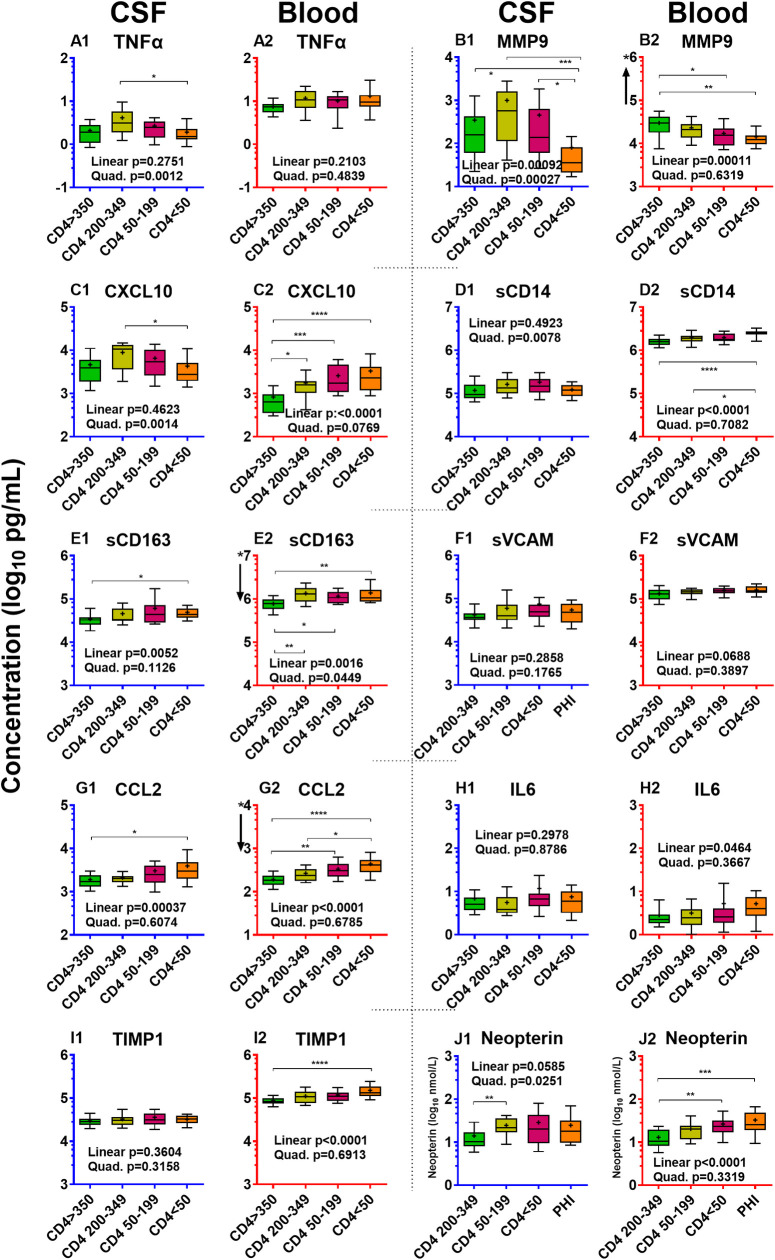
Inflammatory biomarkers in four CD4^+^-defined groups of untreated without HAD. These graphs use the same format, axes and unit scales as **Fig 1** but isolate the results comparing the four NA groups with progressive blood CD4 T cell loss. P values for linear and quadratic trends analysis (also given in **Table 4**) are shown in the text at the bottom of each graph, while the horizontal bars show significant comparisons between groups using Kruskal-Wallis with Dunn’s multiple comparison tests: * P <0.05; ** P <0.01; *** P <0.001; and **** P <0.0001. These figures are extracted from **Fig 1** (which can be referenced for group subject numbers) for visualization of changes and their isolated statistical comparison.

To focus on the changes in CSF inflammatory biomarkers in the face of chronic untreated infection and progressive immunosuppression in the absence of overt HAD, we separately examined the four viremic NA groups defined by blood CD4+ counts; these are shown in isolation in **Fig 4** extracted from **Fig 1** with statistical comparisons added. This isolation emphasizes the varying patterns of biomarker change across the four CD4+ strata. Some first rose before falling, exhibiting an inverted ‘U’ pattern, while others gradually increased or remained relatively static across the four groups.

To more formally test for significant inverted ‘U’ shaped or linear relationships observed visually with descending blood CD4+ counts, we applied linear and quadratic trends analysis to CSF and blood inflammatory biomarkers and to other salient background variables in these four groups (**Table 4**). These analyses confirmed statistical differences in the patterns of changes among the CSF biomarker concentrations. Notably, the four biomarkers with the early rise in PHI—TNFα, CXCL10, sCD14 and neopterin—along with MMP9, exhibited significant inverted ‘U’ patterns with a peak in the midrange CD4 groups followed by decline as blood CD4 cells fell to <50 cells/μL (**Table 4**). Two other markers, sCD163 and CCL2, showed significant linear increases with declining CD4 cells. VCAM, IL6 and TIMP1 did not show either type of trend with CD4 decline. CSF MMP9, in addition to the inverted ‘U’ quadratic trend, exhibited a significant linear *decrease*, with the concentration in the CD4 <50 group lower than the CD4 >350 group.

**Table 4:** Analysis of trends in the four groups of untreated without HAD. The table list the P values solving for quadratic and linear trends for the CSF and blood inflammatory biomarkers and selected background biomarkers over the 4 subject groups defined by blood CD4+ T cell counts shown in **Fig 4**.

VARIABLE		Trends Analysis*	
		Quadratic	Linear
Inflammatory			*P =*
***TNF*α**	***CSF***	**0.0012**	0.2579
	***Blood***	0.4839	0.2103
***MMP9***	***CSF***	**0.0003**	**0.0009**
	***Blood***	0.6319	**0.0001**
***CXCL10***	***CSF***	**0.0014**	0.4623
	***Blood***	0.0769	**<0.0001**
***sCD14***	***CSF***	**0.0078**	0.4923
	***Blood***	0.7082	**<0.0001**
***sCD163***	***CSF***	0.1126	**0.0052**
	***Blood***	0.0449	**0.0016**
***sVCAM***	***CSF***	0.1765	0.2858
	***Blood***	0.3897	0.0688
***CCL2***	***CSF***	0.6074	**0.0004**
	***Blood***	0.6785	**<0.0001**
***IL-6***	***CSF***	0.8786	0.2978
	***Blood***	0.3667	**0.0464**
***TIMP-1***	***CSF***	0.3158	0.3604
	***Blood***	0.6913	**<0.0001**
***Neopterin***	***CSF***	**0.0251**	0.0585
	***Blood***	0.3319	**<0.0001**
**Background**			
***HIV RNA***	***CSF***	**0.0002**	0.9161
	***Blood***	0.4534	**<0.0001**
***WBC***	***CSF***	**0.0148**	**0.0130**
***Alb ratio***	***CSF*:*blood***	0.4740	0.6893
**Neuronal**			
***NFL***	***CSF***	**0.0432**	**<0.0001**
***t-tau***	***CSF***	0.8267	0.2882
***QNPZ-4***	***NP Testing***	0.1670	**0.0016**

*The significance values for each of the inflammatory biomarkers and selected background biomarkers using linear trend analysis solving for linear and quadratic trends.

These patterns of change allow definition of two different inflammatory group patterns which we refer to in the ensuing discussion as *pathogenetic vectors* in the evolution of CNS/CSF infection and CNS pathology (see below). These links are supported by associations of different biomarkers with CSF HIV-1 RNA and WBC counts, on the one hand, and with neurological injury indicated by CSF NfL, on the other. Thus, both CSF HIV-1 RNA and CSF WBC counts shared the inverted ‘U’ pattern of change with the TNFα, MMP9, CXCL10, sCD14 and neopterin group of biomarkers, while CSF NfL and QNPZ-4 testing followed a linear trend of worsening with CD4+ T-cell loss over these four non-HAD groups like CSF sCD163 and CCL2. We should also note that CSF WBCs also showed some linear trend while NfL exhibited a minor quadratic component, suggesting more complexity and elements of mixed associations of these trends. In contrast to the different trends among the CSF biomarkers, in general the blood inflammatory biomarkers exhibited linear increase (CXCL10, sCD14, sCD163, CCL2, IL6, TIMP1 and neopterin), linear decrease in the case of MMP9, or non-significant (TNFα, sVCAM1) trends with CD4 decline across these four subject groups.

### Neuronal injury and CSF inflammation

The CSF inflammatory biomarker concentrations in the HAD group stood out conspicuously from the other subject groups. Concentrations for several were the highest of any group (e.g., TNFα, sCD14, sCD163, sVCAM-1, CCL2, IL6, TIMP1, and neopterin) or comparable to the highest levels in one of the CD4-defined groups (MMP9 and CXCL10) (**Fig 1A**), and all 10 were higher in the HAD group than the untreated groups with comparable blood CD4+ T cell counts (the <50 and 50–199 CD4 groups). This contrasted with the blood inflammatory biomarkers that did not appreciably increase in HAD compared to the comparable blood CD4+ groups.

**Fig 5:**
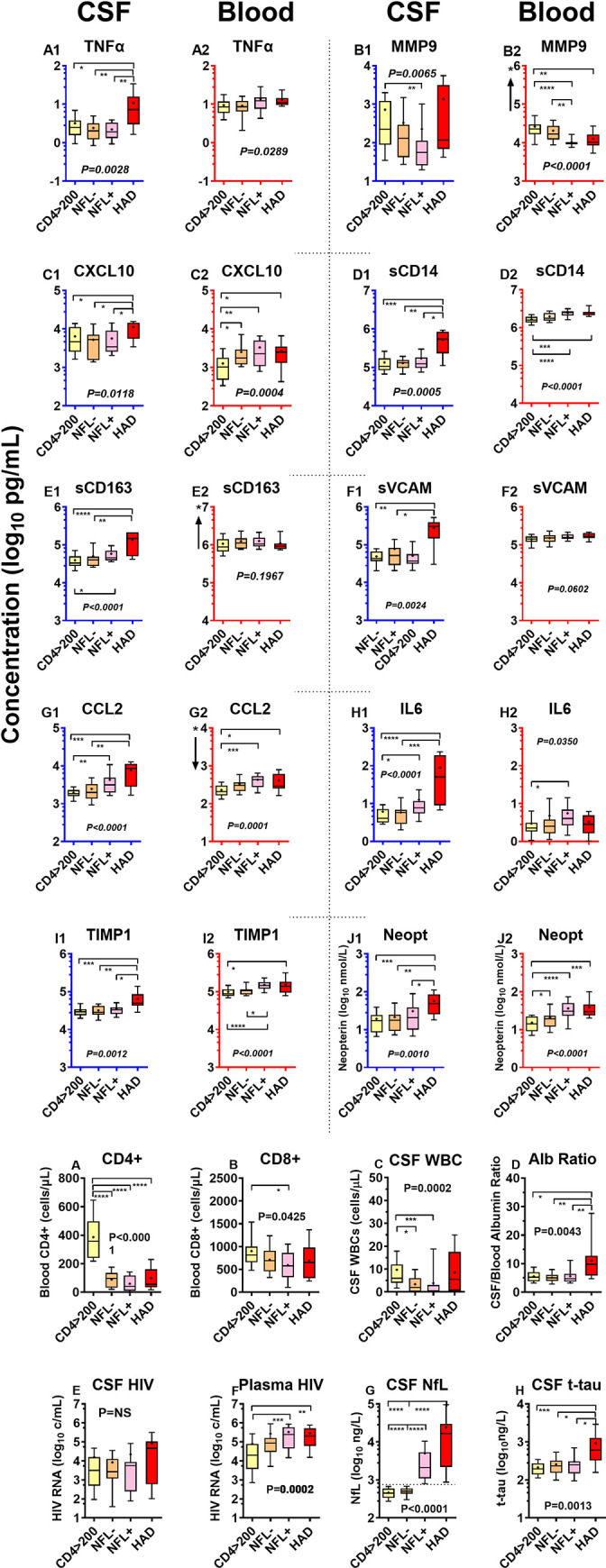
Re-sorted subject groups based on blood CD4 and CSF NfL concentrations. ***a*. *CSF and blood inflammatory biomarkers*.** The panels graph group values for each of the Inflammatory biomarkers in CSF and blood after the subjects were regrouped on the basis of blood CD4+ T cell counts, CSF NfL and HAD diagnosis as described in the text. These graphs use the same format, axes and unit scales as **Fig 1A**, but since the group compositions for the three non-HAD groups are different from the original groups, their colors have been changed. The overall significance of the differences among groups by Kruskal-Wallis are shown by P values in text, while significant differences between groups by Dunn’s multiple comparison test are shown by the horizontal bars: * P <0.05; ** P <0.01; *** P <0.001; and **** P <0.0001. In this analysis the number assayed for CSF and blood inflammatory markers were not fully concordant because of sample shortfalls in some cases. The following list provides the group numbers for CSF and for blood (in parentheses) analyzed for inflammatory markers: CD4 > 200 33 (36); NFL- 17 (17); NFL+ 22 (21); HAD 10 (12). ***b*. *Background biomarkers***. The 8 panels include the same four re-sorted groups with respect to salient background biomarkers. The CD4 counts (A) of the two middle groups, <200 cells/μL were notably also not different from the HAD group, while the CD8 counts (B) also did not differ across these same three groups. The CSF WBC counts were lower in the two NFL groups than the CD4> 200 (C), while the albumin ratio (D) was elevated in the HAD group but similar (and normal) in the other 3 groups. CSF HIV RNA concentration did not statistically differ across the 4 groups (E) but plasma HIV was higher in the HAD and NFL+ groups. By definition, CSF NfL (G) was higher in the NFL+ group and even higher in the HAD group, while t-tau was only elevated in the HAD group (H).

To provide further perspective on the associations of the 10 biomarkers with neural injury, we focused not only on the HAD group defined by their clinical neurological presentation, but also on a second ‘type’ of injury identified by CSF NfL concentrations above the age-corrected norm [38] within the chronic infection groups defined by CD4+ T-cell strata. This injury was common in the groups with 50–199 and <50 CD4+ T-cells/μL (40% and 70% abnormal, respectively). In order to examine differences between this injury and that of subacute HAD and to also compare both of these groups to untreated PLWH without active injury, we re-sorted the untreated subjects with chronic untreated viremia into four groups as previously described [41]: ***1***) subjects with blood ***CD4 >200*** cells per μL and normal age-adjusted NFL (thus combining the initial >350 and 200–350 groups but excluding four with elevated NFL), total subject N = 36; ***2***) those from the two groups with <200 CD4 counts (<50 and 50–199 cells/μL) with CSF NfL concentrations below the age-adjusted laboratory cutoff (designated ***NfL-negative*** or ***NfL-*** total N = 18); ***3***) those from the same two lower CD4+ groups with elevated CSF NfL (designated ***NfL positive*** or ***NfL+*** total N = 22); ***4***) the subacute HAD group as initially defined by their clinical presentation (***HAD***, total N = 12). The results of this re-sorting are shown in **Fig 5**.

This regrouping helps to highlight some of the salient differences between the more highly inflammatory CSF profile in HAD compared to less-inflammatory injury in the NFL+ group.

#### HAD: Highly inflammatory CNS injury

After the regrouping of the chronically infected individuals, the median CSF concentrations of all inflammatory biomarkers except MMP9 were higher in subacute HAD than in the two groups without elevated CSF NfL (i.e., the CD4 >200 and the NfL- groups), again emphasizing the broad character and high level of inflammation associated with clinically overt HAD. Concentrations of some of the CSF inflammatory biomarkers, including TNFα, CXCL10 and sCD14, were higher in the HAD group than in all three of the other groups which did not differ among themselves (**Fig 5A**). Thus, the high level and breadth of the inflammation in the HAD group add to its pathobiological distinction from other types of CNS injury and enforce the clinical diagnostic differentiation of this condition, joining with abnormal t-tau and CSF/blood albumin ratios (and lower CSF αAPP and β levels reported previously [41, 87]).

#### NFL+ group: CNS injury with restricted inflammation

The regrouping segregated individuals with the second type of HIV-related CNS injury (the NFL+ group) from the HAD group. In general, the NFL levels in this group were lower than in HAD but overlapped with its lower quartile (**Fig 5A**). The QNPZ-4 performance was also not as severely impaired (not shown), and the CSF t-tau concentrations and BBB permeability were normal in this group (**Fig 5B**). The CSF inflammatory profile in the NFL+ group was also very different from HAD. CSF levels of TNFα, CXCL10, sCD14, TIMP1 and neopterin were distinctly lower than those of HAD and, importantly, not significantly different from those of the NFL- group and, indeed, also the >200 group, suggesting that these biomarkers do not associate with the neural injury in this NFL+ group. By contrast, CSF sCD163, CCL2, and IL6 were intermediate between HAD and the NFL- group supporting a possible association with this type of injury (**Fig 5A**). Additionally, *blood* concentrations of sCD14, CCL2, TIMP-1 and neopterin also were intermediate or nearer to those of HAD than the groups without elevated NfL, further raising the question of an association of systemic inflammatory responses with this type of injury. These distinctions in both CSF and blood further define a profile of this second type of *low-inflammatory injury* and its distinction from the high-inflammation character of subacute HAD.

### Model of evolving CNS inflammation and the three pathogenic vectors

**Fig 6:**
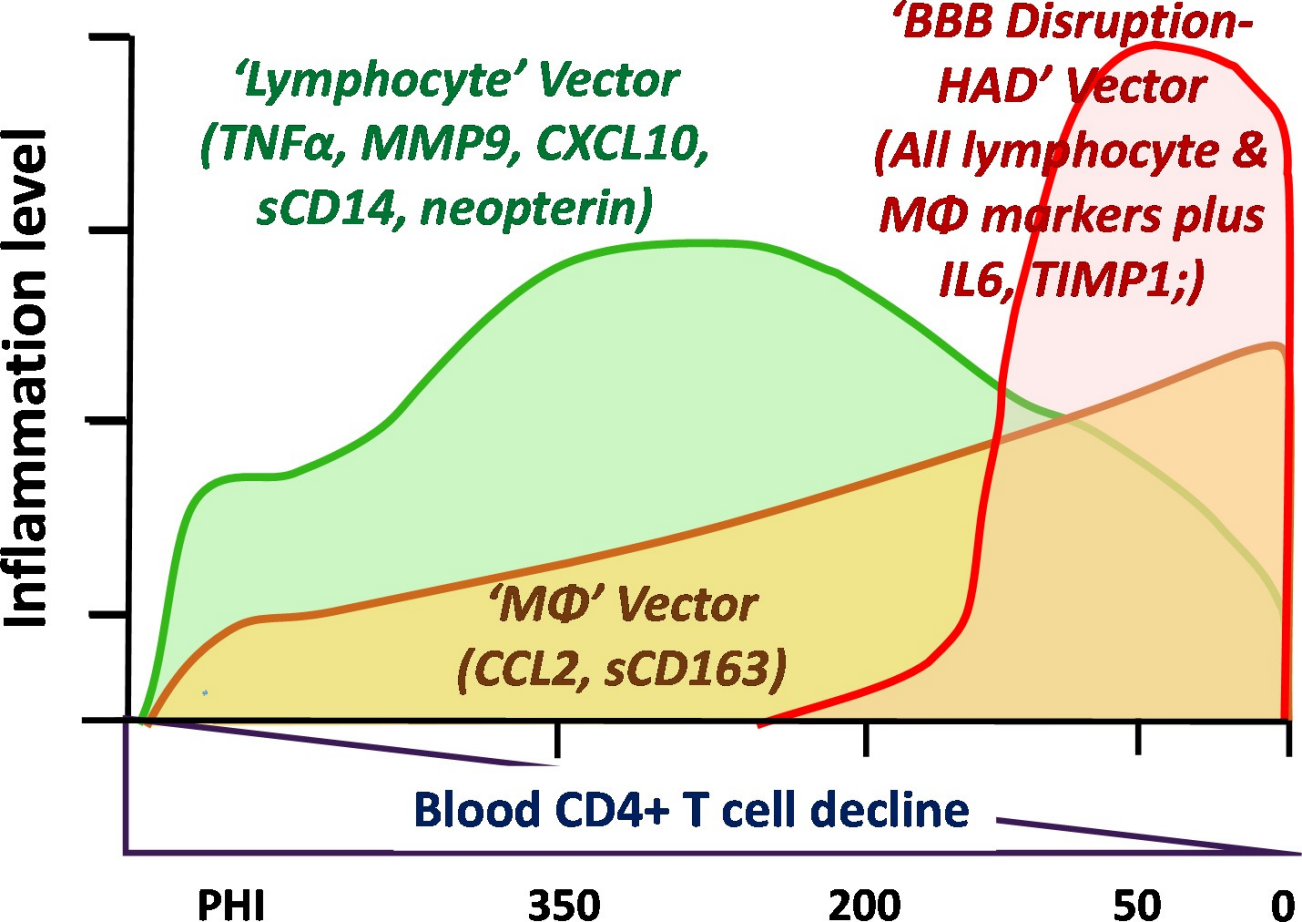
Model of evolving CNS inflammation with systemic and CNS disease progression. This simplified diagram outlines a model of the changes in three components, here referred to as ‘vectors’, of CSF inflammation identified in this study. Because of the variability and relatively small sample, these are not precisely mapped but only roughly outlined to provide a general conceptual framework. The *lymphocyte vector* develops during primary infection, increases as blood CD4+ T cells decline to reach a peak in the middle range of blood CD4+ T-cell counts (centered at 200–350 cells/μL), and then falls as these counts decline further, eventually to levels near normal when the blood CD4+ levels are below 50 cells/μL. This pattern is defined by the quadratic pattern in trends analysis and prominently includes the CSF markers listed in the figure. Importantly, it is also the pattern of CSF WBCs and HIV-1 RNA. The second pattern, the ‘macrophage’ (MΦ) vector, involves a gradual increase in CSF biomarker concentrations with falling blood CD4+ T-cell count. It includes the CSF markers with linear pattern listed in the figure along with a component of CSF MMP-9 and perhaps CSF neopterin. This is also the pattern of CSF NfL concentrations, and thus at a certain threshold this vector appears to associate with the ‘noninflammatory’ type of CNS injury. The third vector includes the disruption of the blood-brain barrier and increased concentrations of all the inflammatory markers in HAD. The cause of this disruptive increase in the barrier is not certain but it associates not only with an increase in all CSF biomarkers, but with more severe CNS injury, higher CSF NfL and elevated levels of CSF t-tau.

The analysis of the 10 CSF biomarkers and the differences among the subject groups provides the basis for proposing a tentative ‘model’ of CNS inflammatory factors in relation to other features of CNS infection and injury. **Fig 6** provides a schematic of this model with three principal *vectors* (for want of a better term) that are active or predominate during different phases of progressive infection and CNS injury.

#### Lymphocytic inflammatory vector

This phase begins during PHI but is most clearly defined in the four CD4+ groups in which lymphocytic CSF biomarkers exhibited an inverted U-shaped pattern conforming to a quadratic solution in the trends analysis (**Fig 4, Table 4**). These biomarkers included CSF TNFα, MMP9, CXCL10, sCD14 and neopterin. Notably, they also prominently correlated with CSF HIV-1 RNA, which along with the CSF WBC, exhibited a similar quadratic pattern of change with systemic disease progression (see also **Fig 1B**). CSF CXCL10 and MMP9 showed the highest overall correlation with the CSF WBC count. These observations, along with the known biological functions of these markers, support the hypothesis that a *lymphocytic vector* of CSF inflammation associates with local meningeal HIV-1 replication that develops early in infection, and in the absence of subacute HAD, reaches its peak when blood CD4^+^ counts are between 200 and 350 cells per μL. This is also associated with the pattern of CSF HIV-1 RNA levels, though to what degree these mediators drive lymphocyte entry and local HIV-1 replication or are a response to infection is uncertain. Likewise, it is uncertain why their concentrations were attenuated after further depression of the blood CD4^+^ T cell count below 50 cells per μL While systemic lymphopenia may provide a partial explanation, it is likely an incomplete one, since robust inflammation (including increase in these CSF biomarkers and pleocytosis) was generated in HAD despite similar low blood CD4+ T cell counts. By itself, this early CSF lymphocytic inflammation was associated with limited neuronal injury (and usually normal CSF NfL levels), and, hence, appeared to have *relatively low* direct neuropathic potential. Using traditional terminology, this may most commonly accompany a clinically silent *aseptic meningitis* that is a frequent feature of untreated HIV-1 infection.

#### Myeloid (monocyte-macrophage) inflammatory vector

This second inflammatory component increases progressively with falling CD4+ T cell counts and, in this series, predominated in more advanced systemic disease as blood CD4 cells fell to 200 cells per μL and below. The CSF biomarker elevations associated with this component most notably included two myeloid CSF biomarkers, sCD163 and CCL2, both exhibiting significant linear trends through the range of falling blood CD4+ T cells (**Table 2**). CSF NfL also exhibited this strong linear trend, and these two macrophage biomarkers were among those that correlated well with CSF NfL, along with CSF sCD14 and sVCAM-1. These associations are consistent with the prevalent hypothesis that HIV-1-related neuronal injury is associated with macrophage recruitment into and activation within the CNS [31, 88, 89]. If elevation of these monocyte-associated biomarkers is involved in ‘low-inflammatory’ CNS injury, this begins to increase in frequency when CD4+ cells fall below 200 per μL, Myeloid cells are important in supporting recruitment and replication of M-tropic HIV-1 within the CNS [90–93] and in generation of neuropathogenic signals and toxins [60].

#### Blood-brain barrier disruptive HIV-1 encephalitis vector

The HAD group, albeit represented in this study by a relatively small sample, was distinguished from the NA groups (including from those with increased CSF NfL) by its robust CSF inflammatory profile with the components of the lymphatic and myeloid vectors boosted above the levels in the non-HAD groups with comparable blood CD4 counts. Indeed, all the CSF inflammatory biomarkers were either frankly higher in HAD than the other groups or nearly so. Among the markers, only the median CSF MMP9 was higher in one or more of the NA groups. Thus, HAD, defined by its subacute clinical presentation, was a broadly and distinctly inflammatory CNS disorder. It was also coupled with other unique features, including common disruption of the blood-brain barrier to albumin as previously reported in a larger case series [82] and elevation of CSF t-tau in addition to NfL [41]. Our data do not define what factors caused the increased CNS vascular permeability or indeed whether it was a cause or consequence of the increased inflammatory milieu and CNS injury. However, HAD presents a distinct enough picture to posit the third pathogenic vector depicted in **Fig 6**.

While we studied blood biomarkers mainly for comparison with CSF and did not include them in this simple model, we also found that some of the blood biomarker changes correlated with CSF NfL, most notably blood neopterin and sCD14. While this may simply be related to CD4+ T-cell loss without direct effect on CNS events, it might also suggest that the character of systemic inflammation predisposes to development of neuronal injury by influencing the character of CNS inflammation or, more directly, by mechanisms that are not yet apparent. Whatever the reasons for the association, elevations of some of these blood inflammatory biomarkers in the NfL+ and HAD subjects may help to distinguish individuals at higher risk of CNS injury.

### Viral suppression: Reduced CSF inflammation in ART-treated and elite controllers compared to viremic individuals

**Fig 7:**
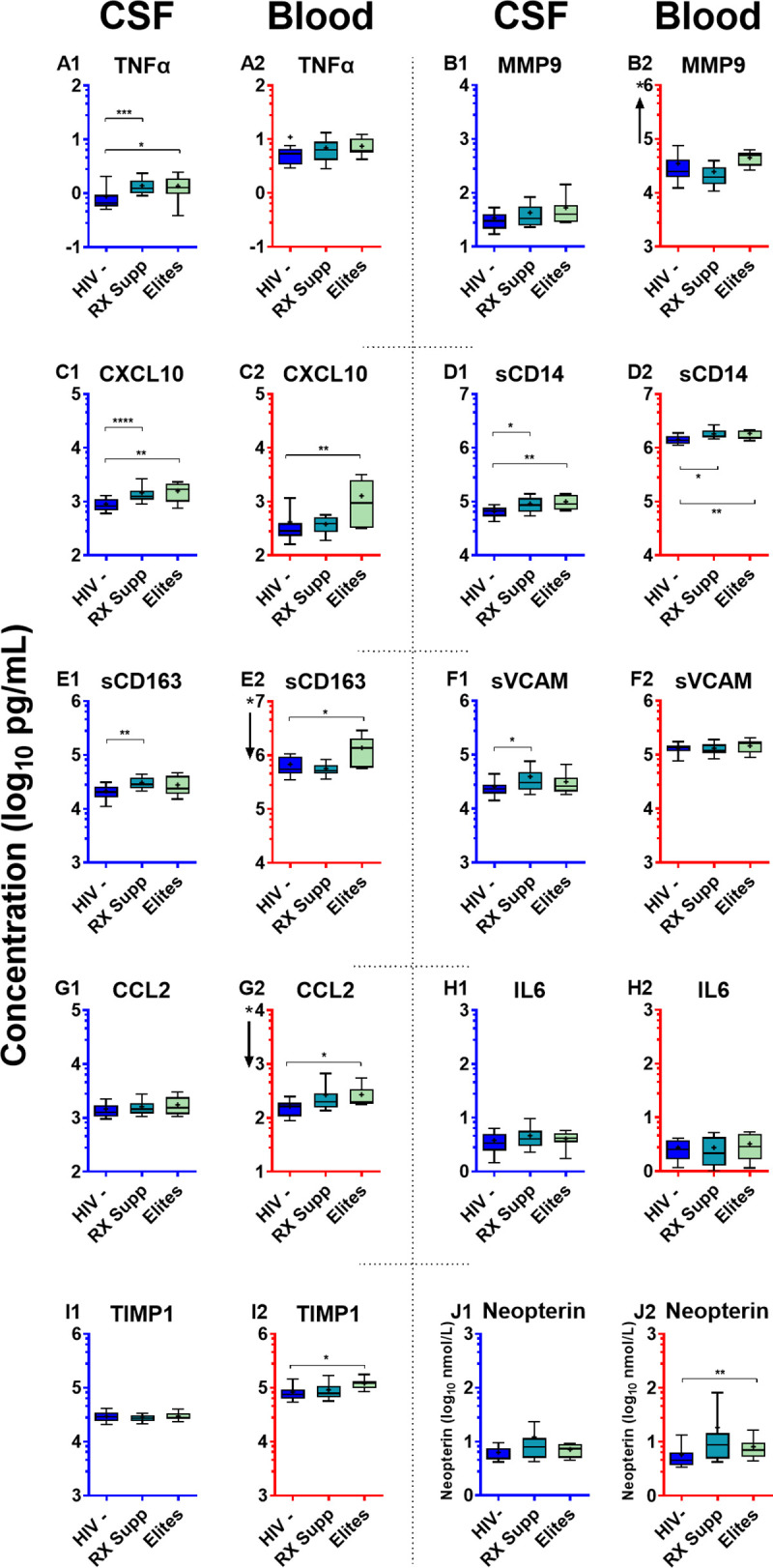
CSF and blood inflammatory biomarkers in ART-suppressed and elite controllers. Inflammatory biomarkers in the two virally suppressed groups are compared to HIV-uninfected controls. These graphs use the same format, axes and unit scales as **Fig 1** but show the isolated data of the HIV negatives, treated-suppressed and elites, with each of the latter two groups compared independently to the HIV negative group with significant differences noted by the horizontal bars: * P <0.05; ** P <0.01; and *** P <0.001. These figures are extracted from **Fig 1** for visualization and isolated statistical comparison. The number of subjects in each group is provided in the legend to **Fig 1**.

Suppressive ART clearly attenuated the HIV-1-related CNS inflammatory response, reducing all the measured CSF inflammatory biomarkers, while elite control similarly was accompanied by reduced inflammation compared to patients with viremia (**Fig 7**).

In neither of the infected groups with viral suppression was the reduction in inflammation ‘complete’, and concentrations of several of the CSF biomarkers were higher than in the HIV-uninfected controls. This was also the case with several of the systemic inflammatory biomarkers that did not fully normalize in these two settings as has also been documented in other studies [40, 52, 53, 65, 94]. Residual CSF biomarker elevations in the ART- suppressed groups included TNFα, CXCL10, sCD14, sCD163 and sVCAM-1, while several others showed elevations that did not reach statistical significance. Among blood biomarkers only sCD14 was significantly elevated in the treated group compared to the HIV negatives, though again mild elevations of other blood biomarkers were present but short of significant differences. In the elite controllers, similar mild elevations were found in some of the CSF biomarkers, including TNFα, CXCL10 and sCD14, while elevated blood levels of CXCL10, sCD14, sCD163, CCL2, TIMP1 and neopterin were present. Thus, the CSF biomarkers included low-level elevations of both lymphocytic and macrophage biomarkers while the blood included elevation of sCD14 with milder elevations of others. These observations raise some essential questions, including: *What is the cause of the persistent CSF inflammation*? *Does it relate to the CNS HIV-1 reservoir provoked by viral gene expression or low-level CNS viral production*, *or to repeated exposure via circulating infected CD4+ T cells*? *What are the consequences of this low-level inflammation for the CNS*? *Does it lead to continued injury and indolently progressive neurological dysfunction or is it wholly part of a neurologically benign or protective response*?

### Limitations of study

This study has a number of limitations. The size of each group was relatively small, and this was especially the case with the HAD and elite groups that were limited by availability. The control group differed in age from some of the infected groups, particularly from those with more advanced HIV-1-infected patients. Importantly, the multiple exploratory comparisons can foster misleading associations or differences. Furthermore, the results have been discussed in terms of disease progression, but it is important to emphasize that this was a cross-sectional rather than longitudinal study, so that we present a longitudinal ‘construction’ rather than a true set of longitudinal observations. For these reasons, our findings and conclusions should be considered provisional, although further studies, particularly using a prospective design, are now difficult because of the salutary effects of early and broad application of ART. Nonetheless, these observations outline an initial framework or scaffold for defining CNS inflammation in the spectrum of HIV-1 infection, albeit one that will benefit from both confirmation and amplification by more focused studies examining larger subject groups and a more extensive array of inflammatory biomarkers.

Additionally, CSF analyte concentrations can reflect complex contributions from several sources, including the brain parenchyma, perivascular zones and leptomeninges, so that the anatomical source of the individual CSF inflammatory molecules measured in these studies likely varied and may have been different among the subject groups and disease stages. Likewise, the cell origins may be multiple and complex, including lymphocytes, myeloid cells, vascular components, astroglia and even neurons. Additionally, the functional impact and mechanistic significance of each of the measurements remain speculative since their *in vivo* roles are largely undefined and likely often pleotropic. Thus, this study provides empirical data and a disease stage framework for pathogenetic interpretation and future exploration but does not directly define either the mechanisms of the observed changes or their impact. While the results show a variety of associations among inflammatory biomarkers, disease stages and other biomarkers, the pathogenetic implications of these associations, particularly implications for causality, remain to be more precisely defined. However, these observations might now be integrated into considerations of particular roles for individual biomarkers, focusing on how they might interact within each clinical HIV stage or setting.

## Conclusions

Using a panel of 10 functionally diverse CSF inflammatory biomarkers these results show that that systemic HIV-1 infection is accompanied by a broad CNS inflammatory state that evolves with a definable pattern through its course: beginning in its first year, changing through progressive CD4+ T cell loss and development of CNS injury, and persisting during viral suppression. Each of the measured biomarkers showed concentration alterations over this course, though with importantly different patterns at the various stages of systemic infection and neurological injury. While the CNS inflammatory responses were indeed diverse, they also exhibited a coherent pattern of evolution suggesting predominance of different *pathogenetic vectors* in different stages or settings. The results provide an empirical framework for further exploration of the underlying interactions of HIV-1, inflammation and CNS perturbation through the course of infection and eventual more precise definition of stage-related mechanisms.

## Supporting information

S1 Table Full data set of the study results. This table contains the raw data underlying this report, including the background data and inflammatory biomarker measurements used in analysis. The age-corrected NfL results highlighted in light red are those above the age-corrected upper limit of normal. These data are presented to facilitate review and further analysis by other investigators.
(XLSX)

S1 File (PDF)

## Glossary of terms and abbreviations

Inflammatory biomarkers: TNFα: tumor necrosis factor alpha
MMP9: matrix metalloproteinase-9
CXCL10: C-X-C motif chemokine 10 (IP-10)
sCD14: soluble monocyte differentiation antigen CD14
sCD163: scavenger receptor cysteine-rich type 1 protein M130)
sVCAM-1: soluble vascular cell adhesion protein 1
CCL2: C-C motif chemokine 2 (MCP-1)
IL6: interleukin-6
TIMP1: metalloproteinase inhibitor 1)
General terms: ADC: AIDS dementia complex
BBB: blood-brain barrier
CSF: cerebrospinal fluid
CNS: central nervous system
HIV-1: human immunodeficiency virus type one
HAD: HIV-associated dementia
CD4 cells: CD4+ T-lymphocytes
WBCs: white blood cells)
NfL: neurofilament light chain protein
PHI: primary HIV infection
PLWH: people living with HIV
QNPZ-4: quantitative neuropsychological performance Z score on four tests
