# Supplementary material for: Compartmentalization of cerebrospinal fluid inflammation across the spectrum of untreated HIV-1 infection, central nervous system injury and viral suppression
Source: PLoS One. 2021 May 13;16(5):e0250987. doi: 10.1371/journal.pone.0250987 (PMC8118251; doi:10.1371/journal.pone.0250987)
Supplement: S1 File — (PDF) [file pone.0250987.s002.pdf]

Fig 1

1a. Inflammatory Biomarkers

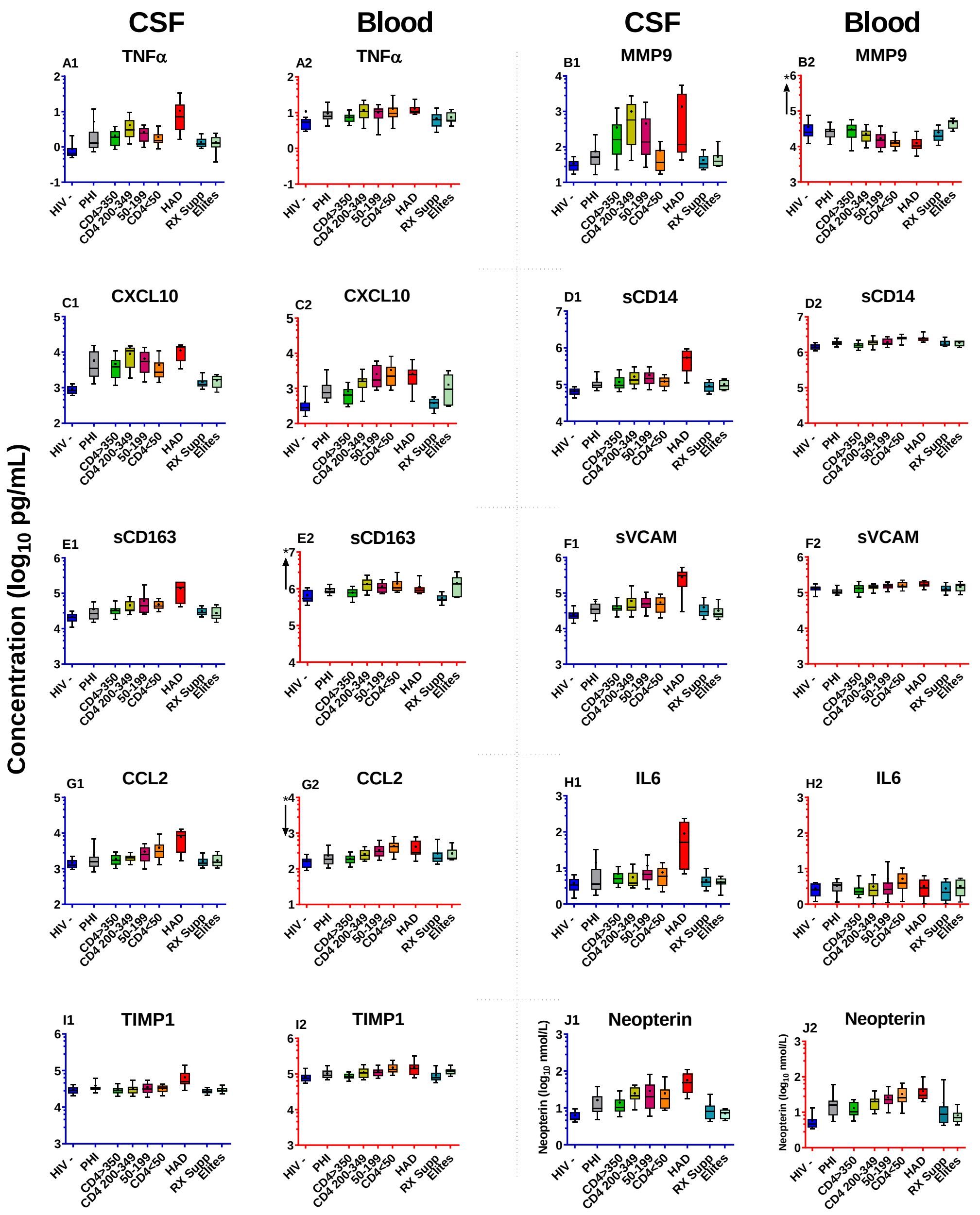

1b. Background Biomarkers

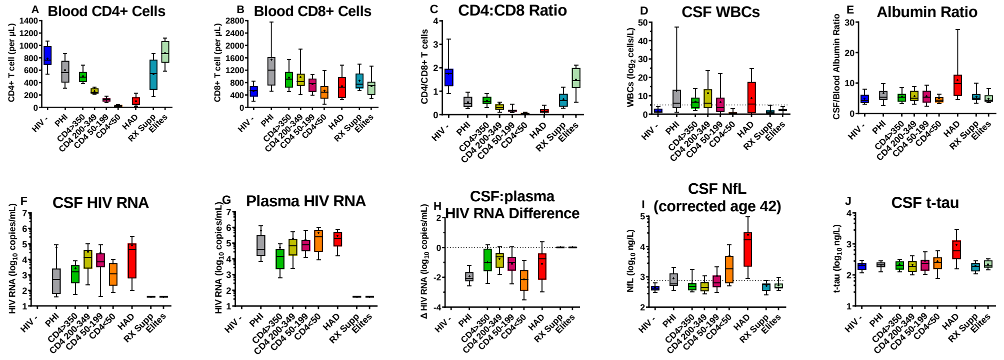

Fig 2

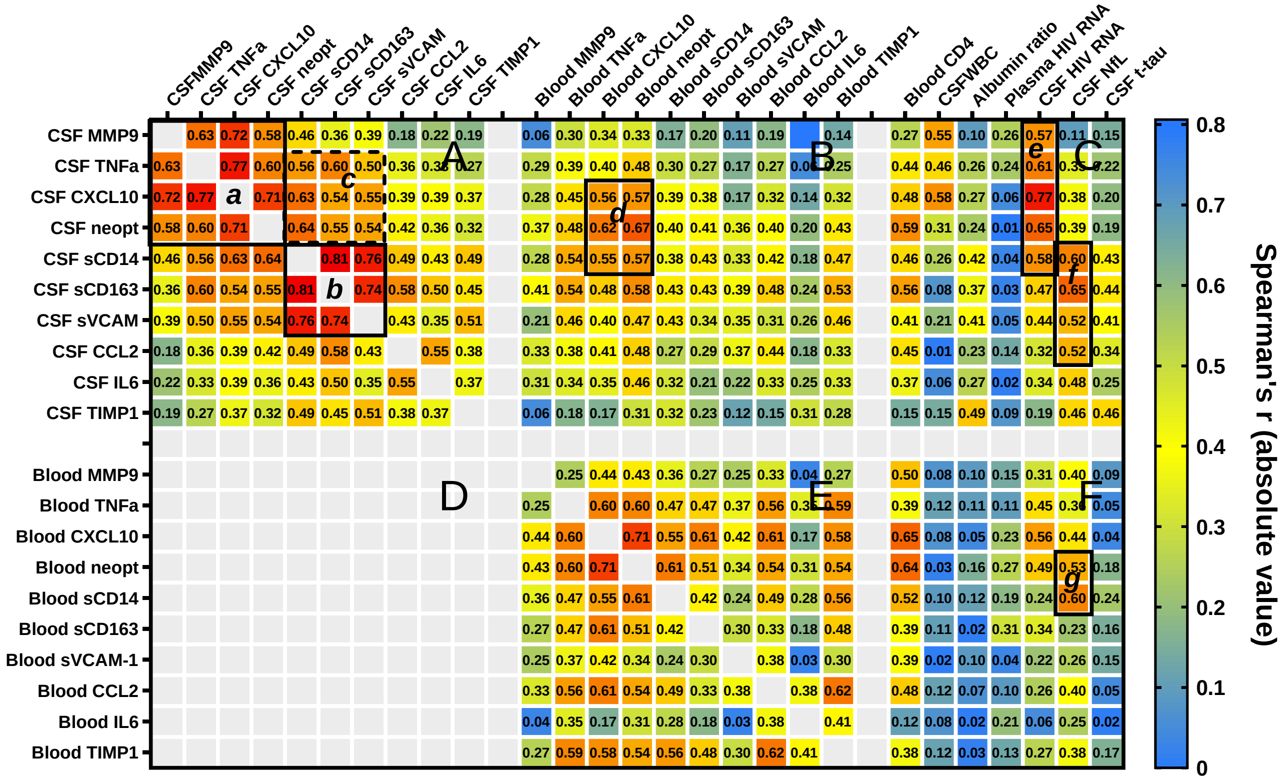

CSFMMP9
CSF TNFa
CSF CXCL10
CSF neopt
CSF sCD14
CSF sCD163
CSF sVCAM
CSF CCL2
CSF IL6
CSF TIMP1
Blood MMP9
Blood TNFa
Blood CXCL10
Blood neopt
Blood sCD14
Blood sCD163
Blood sVCAM
Blood CCL2
Blood IL6
Blood TIMP1
Blood CD4
CSFWBC
Albumin ratio
Plasma HIV RNA
CSF HIV RNA
CSF NfL
CSF t-tau
CSF MMP9
Blood sVCAM-1
A
B
C
D
E
F
a
b
c
d
e
f
g
Spearman's r (absolute value)
0.8
0.7
0.6
0.5
0.4
0.3
0.2
0.1
0

Fig 3

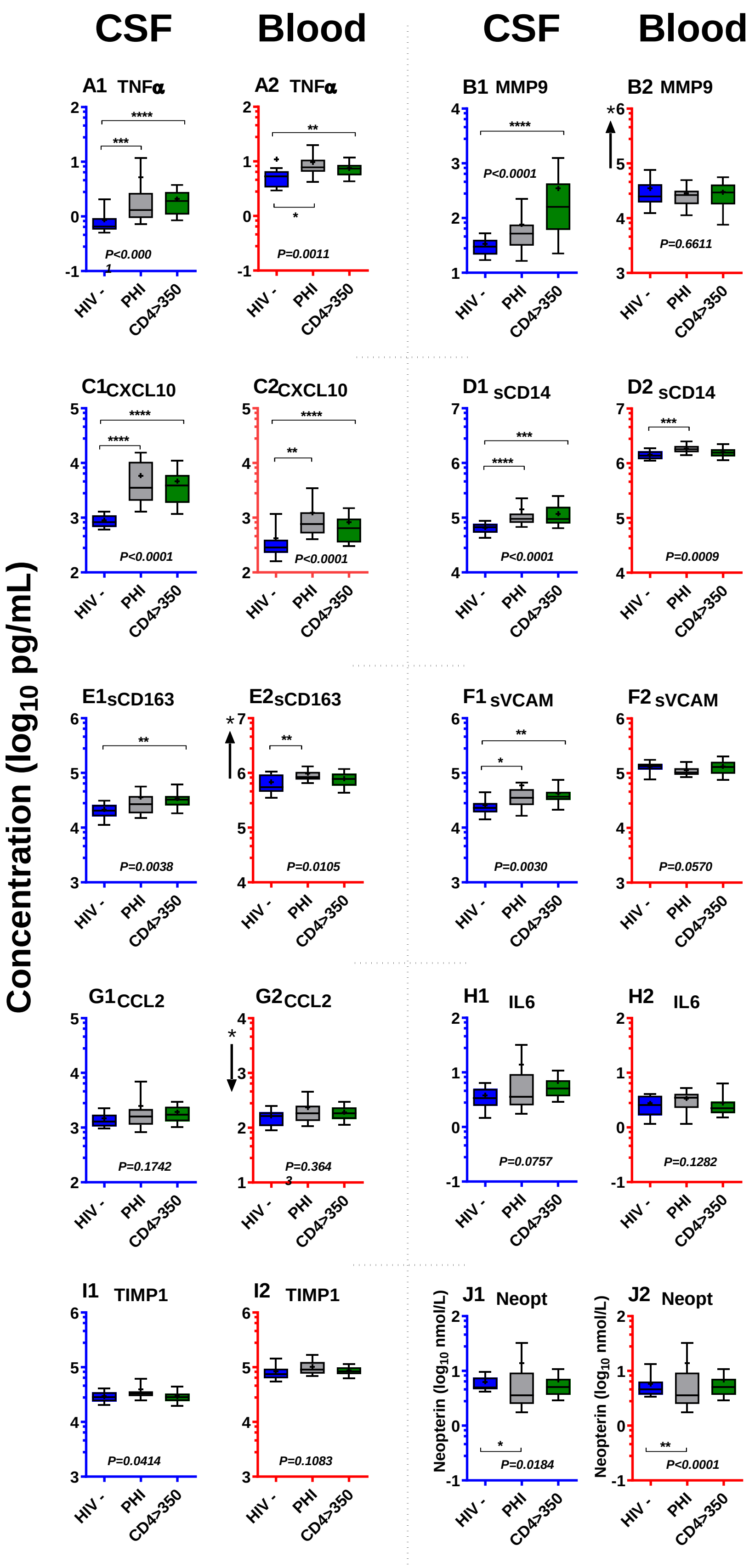
CSF
Blood
CSF
Blood
Concentration (log10 pg/mL)
A1 TNFα
P<0.0001
A2 TNFα
P=0.0011
B1 MMP9
P<0.0001
B2 MMP9
P=0.6611
C1 CXCL10
P<0.0001
C2 CXCL10
P<0.0001
D1 sCD14
P<0.0001
D2 sCD14
P=0.0009
E1 sCD163
P=0.0038
E2 sCD163
P=0.0105
F1 sVCAM
P=0.0030
F2 sVCAM
P=0.0570
G1 CCL2
P=0.1742
G2 CCL2
P=0.3643
H1 IL6
P=0.0757
H2 IL6
P=0.1282
I1 TIMP1
P=0.0414
I2 TIMP1
P=0.1083
J1 Neopt
P=0.0184
J2 Neopt
P<0.0001
Neopterin (log10 nmol/L)
HIV -
PHI
CD4>350

Fig 4

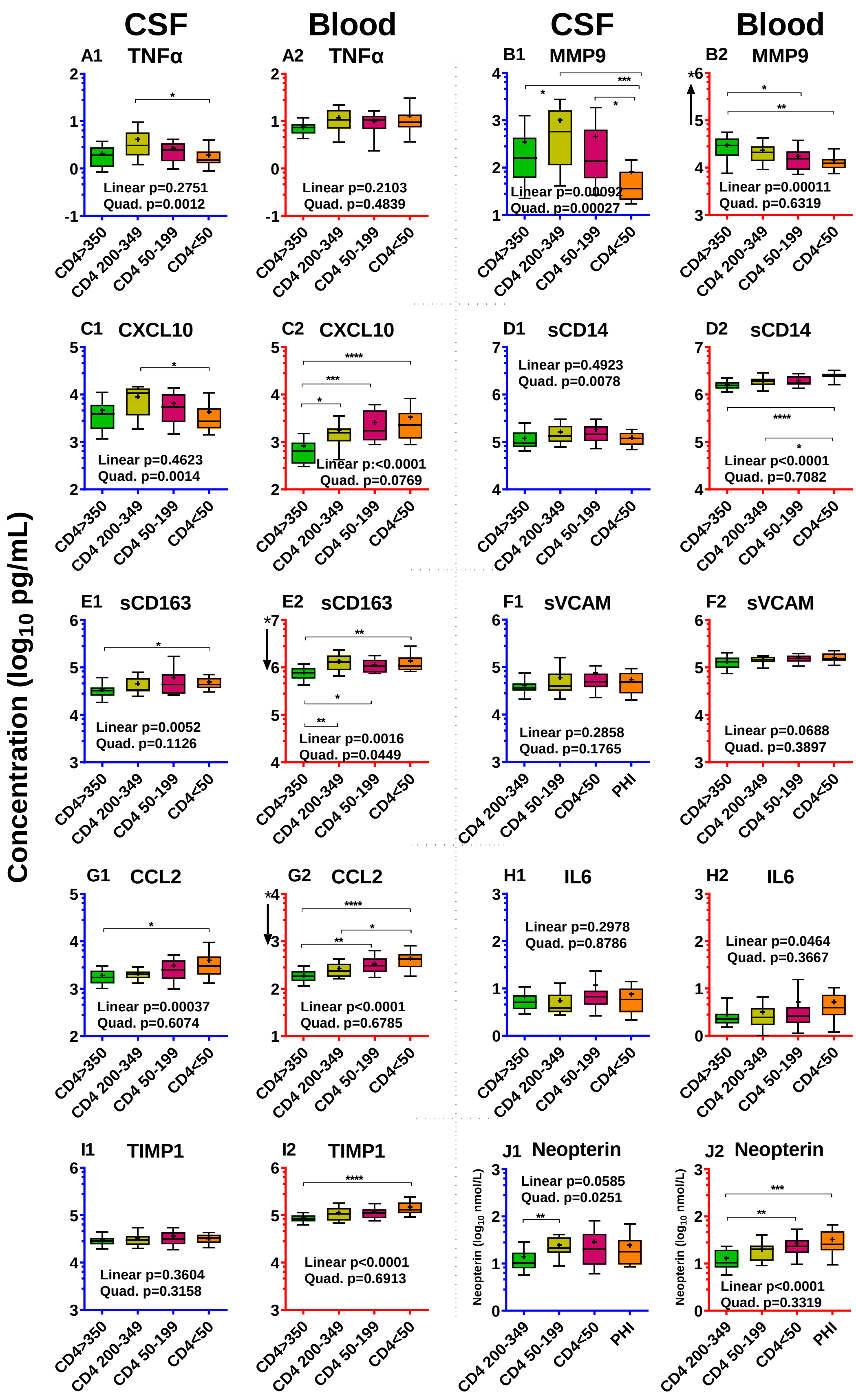
CSF
Blood
CSF
Blood
A1 TNFα
Linear p=0.2751
Quad. p=0.0012
A2 TNFα
Linear p=0.2103
Quad. p=0.4839
B1 MMP9
Linear p=0.00092
Quad. p=0.00027
B2 MMP9
Linear p=0.00011
Quad. p=0.6319
C1 CXCL10
Linear p=0.4623
Quad. p=0.0014
C2 CXCL10
Linear p:<0.0001
Quad. p=0.0769
D1 sCD14
Linear p=0.4923
Quad. p=0.0078
D2 sCD14
Linear p<0.0001
Quad. p=0.7082
E1 sCD163
Linear p=0.0052
Quad. p=0.1126
E2 sCD163
Linear p=0.0016
Quad. p=0.0449
F1 sVCAM
Linear p=0.2858
Quad. p=0.1765
F2 sVCAM
Linear p=0.0688
Quad. p=0.3897
G1 CCL2
Linear p=0.00037
Quad. p=0.6074
G2 CCL2
Linear p<0.0001
Quad. p=0.6785
H1 IL6
Linear p=0.2978
Quad. p=0.8786
H2 IL6
Linear p=0.0464
Quad. p=0.3667
I1 TIMP1
Linear p=0.3604
Quad. p=0.3158
I2 TIMP1
Linear p<0.0001
Quad. p=0.6913
J1 Neopterin
Linear p=0.0585
Quad. p=0.0251
J2 Neopterin
Linear p<0.0001
Quad. p=0.3319
Concentration (log10 pg/mL)
Neopterin (log10 nmol/L)
CD4>350
CD4 200-349
CD4 50-199
CD4<50
PHI

Fig 5

5a. Inflammatory Biomarkers

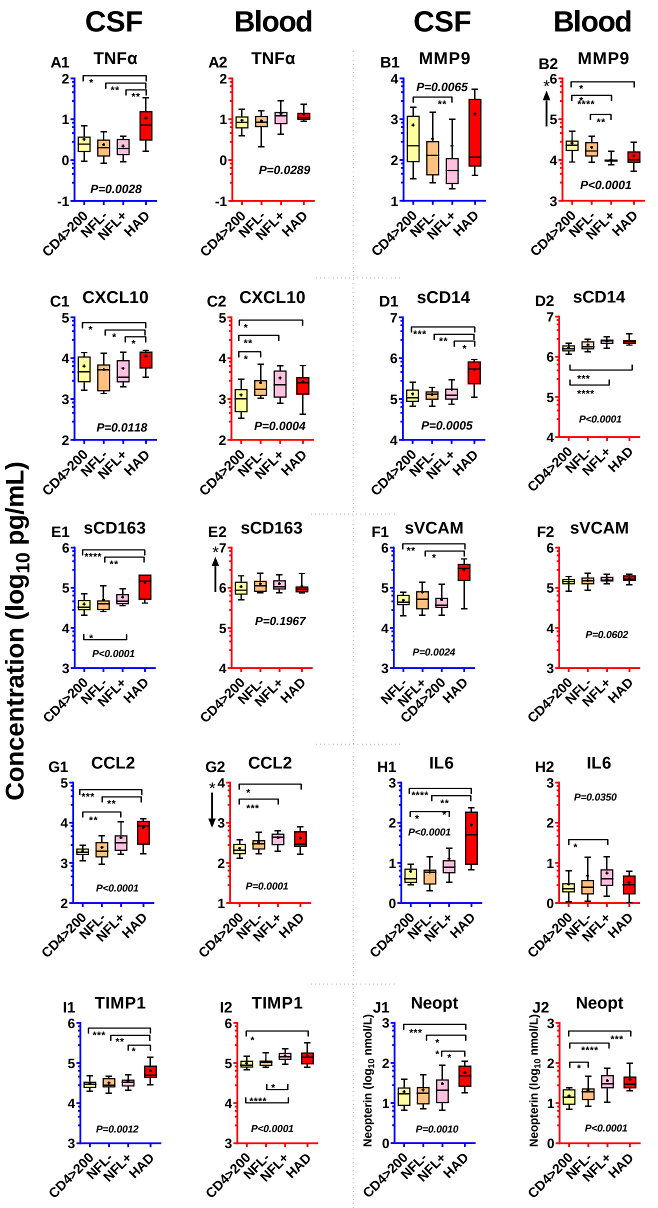

5b. Background Biomarkers

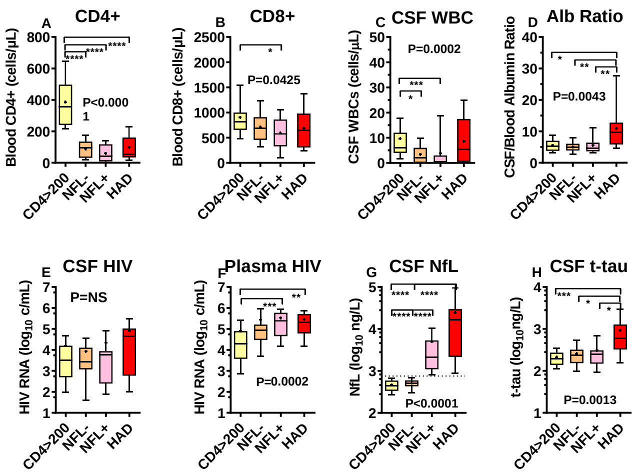

Fig 6

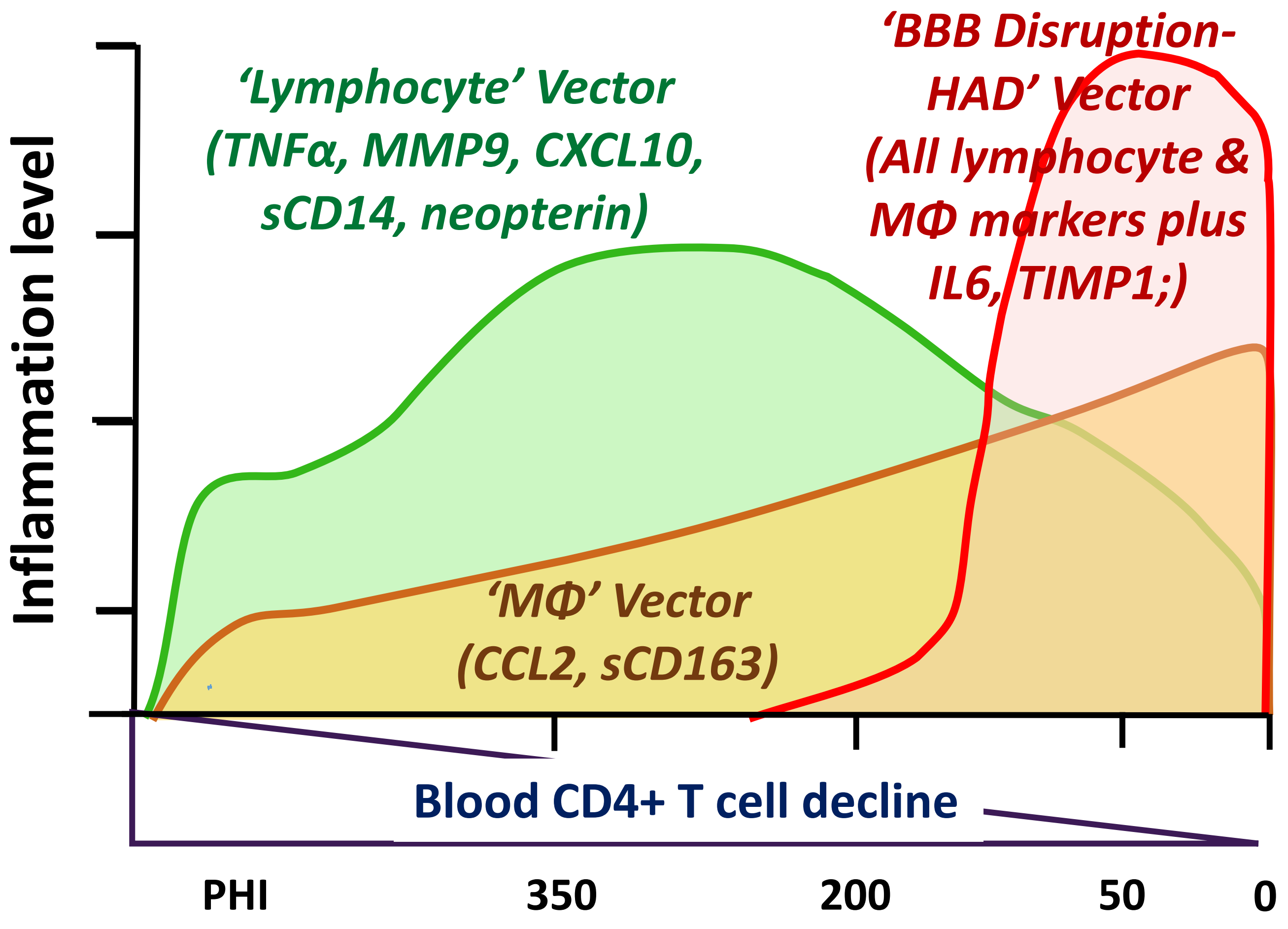

'BBB Disruption-
HAD' Vector
(All lymphocyte &
MΦ markers plus
IL6, TIMP1;)
'Lymphocyte' Vector
(TNFα, MMP9, CXCL10,
sCD14, neopterin)
Inflammation level
'MΦ' Vector
(CCL2, sCD163)
Blood CD4+ T cell decline
PHI
350
200
50
0

Fig 7

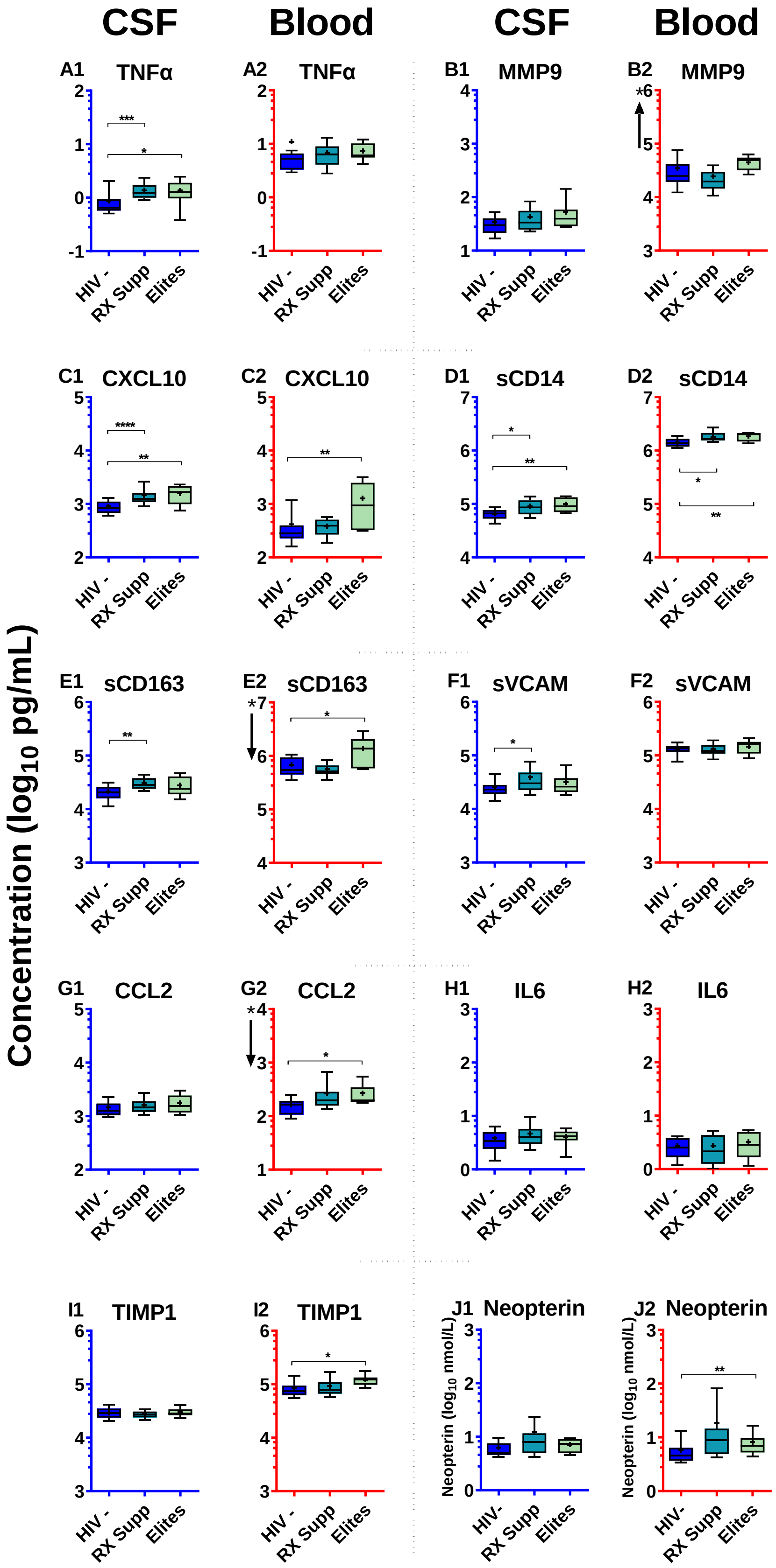
CSF
Blood
CSF
Blood
A1 TNFα
A2 TNFα
B1 MMP9
B2 MMP9
C1 CXCL10
C2 CXCL10
D1 sCD14
D2 sCD14
E1 sCD163
E2 sCD163
F1 sVCAM
F2 sVCAM
G1 CCL2
G2 CCL2
H1 IL6
H2 IL6
I1 TIMP1
I2 TIMP1
J1 Neopterin
J2 Neopterin
Concentration (log10 pg/mL)
Neopterin (log10 nmol/L)
HIV -
RX Supp
Elites
